# Natural Plant-Derived Compounds Targeting Oxidative Stress and Inflammation in NAFLD—Mechanisms and Repositioning Potential

**DOI:** 10.3390/cimb48050465

**Published:** 2026-04-29

**Authors:** Rafailia-Eirini Theodorou, Nikiforos Vrettos, Panagiotis Theodosis-Nobelos

**Affiliations:** Department of Pharmacy, School of Health Sciences, Frederick University, Nicosia 1036, Cyprus

**Keywords:** non-alcoholic fatty liver disease, NAFLD, MASLD, oxidative stress, inflammation, phytochemicals, natural compounds, metabolic syndrome, multi-targeting compounds

## Abstract

Non-alcoholic fatty liver disease (NAFLD) is the most common liver disease, with advanced stages potentially progressing to hepatocellular carcinoma. It is a multifactorial condition associated with metabolic syndrome, diabetes, and hormonal imbalance, leading to metabolic alterations that are intensified by inflammation. An important additional factor that amplifies these effects is oxidative stress, which interacts with inflammatory pathways and contributes to disease progression. This review evaluates evidence from in vitro, in vivo, and clinical studies on widely investigated natural compounds, including cinnamic acid, stilbene and quinone derivatives, coumarinoids, tannins, and miscellaneous phenol-containing compounds and alkaloids, focusing on their antioxidant, anti-inflammatory and multi-functional properties. These compounds have demonstrated beneficial effects such as reduction of lipid accumulation, improvement of insulin resistance, modulation of inflammatory cytokines (e.g., TNF-α, IL-6), and attenuation of oxidative stress markers, with several studies reporting improvements in liver enzymes and histological features of steatosis. The aim is to assess their potential to improve NAFLD beyond their established biological activities and to explore their repositioning potential as multi-targeted agents for complementary or second-line therapeutic strategies. Their plant-derived origin and broad therapeutic profiles suggest a favorable safety margin. However, further well-designed clinical studies are required to better define their efficacy, optimal dosing, pharmacokinetics and safety, as well as to clarify their mechanisms of action and their potential role in NAFLD management.

## 1. Introduction

Among the liver diseases, non-alcoholic fatty liver disease (NAFLD) appears to be the most common chronic disease of the liver, with its pathology ranging from simple steatosis and lipid accumulation (≥5%), up to non-alcoholic steatohepatitis (NASH) with fibrosis and cirrhosis, and at more advanced stages, to hepatocellular carcinoma (HCC) [1,2]. Thus, apart from increased risk for liver damage, NAFLD is related with severe extrahepatic complications, including cardiovascular disease, diabetes, kidney and colorectal dysfunction and hormonal deregulation conditions, such as hypothyroidism, polycystic ovary syndrome and other endocrinopathies [3,4]. This relation seems to be ambivalent since NAFLD progression has as its main risk factors obesity, diabetes mellitus, metabolic abnormalities and endocrine disruption, together with aging and microbiome, resulting in being a metabolic manifestation of genetic and epigenetic triggers [5,6,7].

**Figure 1:**
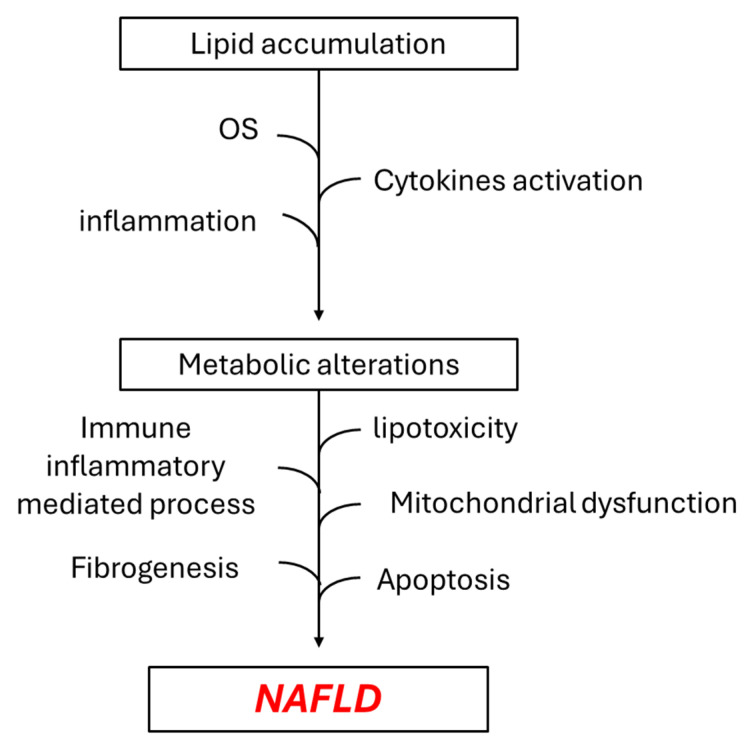
Factors intervening in NAFLD progression.

The pathophysiology of NAFLD is multifactorial (“multiple hits”), with lipid accumulation being the main first hit, causing metabolic alterations that are intensified via the inflammation and cytokines activation, together with oxidative stress progression (those two are considered as the second hit), whilst various other factors related or partly related to these conditions may further assist NAFLD progression via increased lipotoxicity and immune-mediated inflammatory processes, with mitochondrial dysfunction, apoptosis and fibrogenesis among them [8,9,10] (Figure 1).

Until nowadays, current treatment options of NAFLD rely on dietary and lifestyle management with physical exercise application, whilst the pharmacological approaches of existing drugs concern mainly antidiabetic, hypolipidemic, cellular protective and anti-obesity agents [11,12]. However, only two of them (pioglitazone and vitamin E) have been proposed, after evidence-based guidelines, for clinical practice in selected patients [13]. Thus, the safety and efficacy of several monotherapies and/or drug combinations targeting various mechanisms of NAFLD are currently being evaluated in vitro and in vivo in animal and human studies, with promising, in many cases, results on the reversal of the parameters of the disease and its comorbidities. In view of these signs and taking into consideration the multifactorial nature of NAFLD, and that oxidative stress plays a crucial role in its progression, in this review we will try to analyze the impact of oxidative stress and inflammatory processes in this disease and analyze the potential of naturally derived antioxidant compounds or extracts of different nature and indications (with the exception of the wide category of flavonoids) that may be of assistance towards its manipulation, also exploiting their multi-functional potential.

It should be noted that the terminology of non-alcoholic fatty liver disease (NAFLD) has evolved in recent years. The term metabolic dysfunction-associated fatty liver disease (MAFLD) was initially proposed to better reflect the central role of metabolic dysregulation [7,14]. More recently, an international multi-society consensus introduced the term metabolic dysfunction-associated steatotic liver disease (MASLD) to provide a more inclusive and clinically relevant definition [15,16]. In the present review, the term NAFLD is primarily used, as it remains widely adopted in the majority of the cited literature; however, it is conceptually aligned with MAFLD/MASLD, and the findings discussed are applicable across these updated definitions.

The literature search for this review was conducted using three major scientific databases, PubMed, Scopus, and Google Scholar, in order to ensure broad coverage of biomedical, pharmacological, and interdisciplinary research. The search strategy included combinations of keywords such as “NAFLD”, “MASLD”, “MAFLD”, “oxidative stress”, “inflammation”, “natural compounds”, and “plant-derived antioxidants”. Inclusion criteria comprised original research articles (in vitro, in vivo, and clinical studies) and relevant reviews that investigated the antioxidant and/or anti-inflammatory effects of natural compounds in the context of NAFLD (MASLD/MAFLD). Studies were selected based on relevance to the topic, methodological rigor, and clarity of reported outcomes, while articles lacking direct association with NAFLD pathophysiology or without mechanistic insight were excluded, ensuring a comprehensive and focused synthesis of current evidence.

### The Role of Oxidative Stress and Inflammation in the Pathogenesis of NAFLD

Triglycerides and saturated fatty acid accumulation in the liver have been related to NAFLD provocation, increasing lipid peroxidation and oxidative stress with inflammation and fibrosis induction [17,18]. These effects of lipids may rely on their direct toxic effects on the cells or on their indirect lipotoxic intermediates and the activation of kinases and of mitochondrial and endoplasmic reticulum stress [18,19]. The produced ROS lead to peroxidation of the cellular membranes and of the lysosomal membrane with parallel protease release and Ca^2+^ leakage from the ER, with induction of release of cytochrome C and deregulation of mitochondrial energy production and electron transfer leading to apoptotic signals and to NADPH oxidase (NOX)-derived ROS formation [20,21,22]. In addition to saturated fatty acids, excessive cholesterol could also lead to lipotoxicity, intensifying the sensitizing of the hepatic cells to inflammatory, damaging and apoptotic signals, with glutathione (GSH) depletion and lipid droplet formation that activate inflammasome and Kupfer and macrophage cells in the liver, with an increase in IL-1β and TNF-α (interleukin-1β and tumor necrosis factor-α) [23,24,25]. In metabolic imbalance and redox disorders, apart from metabolites (FFAs, oxidized lipids and mitochondrial DNA), pattern recognition receptors (PRRs) [Toll-like receptors (TLRs) and nucleotide-binding oligomerization domain-like receptors (NLRs)] are activated, giving rise to immune reaction signaling processes [26,27]. Their activation can lead to synchronized inflammatory and oxidative injury via activation of enzymes like NOX and pro-inflammatory genes, which, together with the advanced glycation end-products (AGEs), derived by the oxidation of sugars and their conjugation with cytoplasmic proteins, could increase the cellular toxicity in general and the progression of NAFLD [28,29,30]. In combination with the direct debilitating role of AGEs, they can also activate their receptors (RAGE), which are a PRR, too, with their oxidation by ROS leading to their stimulatory effect on NOX1 and the production of more ROS, together with the increased production of reactive species during the AGE formation process [31,32]. Furthermore, ROS seem to stimulate NLRP3 (NOD-like receptor protein 3) inflammasome formation and maturation, with caspase 1 activation and pro-inflammatory cytokine maturation (IL-1β and IL-18), whereas endogenous antioxidant enzymes such as catalase seem to reduce this activation [33,34].

**Figure 2:**
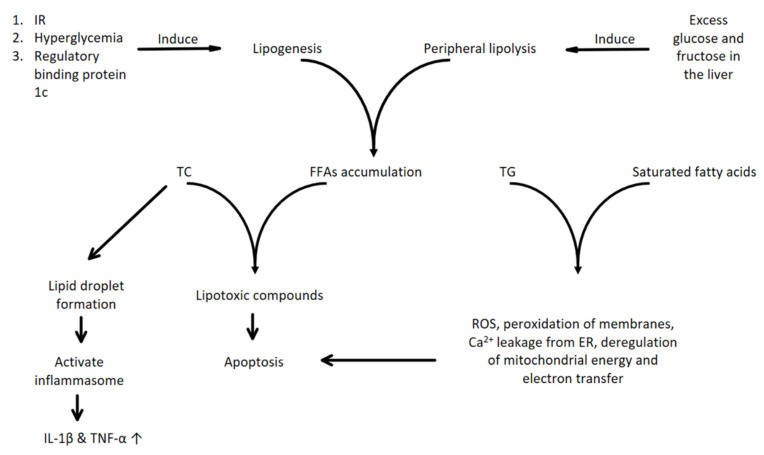
Lipotoxicity and metabolic imbalance activate inflammatory and oxidative pathways in NAFLD.

The two main sources of FFAs accumulated in the liver are derived from peripheral lipolysis, which is highly controlled by insulin, and the second by de novo lipogenesis (DNL) due to excess glucose and fructose levels in the liver [12,35]. Thus, high insulin levels or resistance and hyperglycemia may lead to activation of carbohydrate element binding protein and the sterol regulatory element binding protein 1c, which induce the expression of lipogenic genes, increasing even more the hepatic synthesis of FFAs from sugars and adding to the increased lipid content by DNL. This further deteriorates the NAFLD and produces a vicious cycle of lipid deregulation [36,37]. If this balance in lipids is compromised, FFAs can partially be converted to lipotoxic compounds, such as ceramides, diacyloglyceroles and lysophosphatidylcholine species, promoting the inflammatory metabolic stress of the hepatic cells and their apoptosis [12,38] (Figure 2). Additionally, FFA accumulation promotes metabolic and hormonal signaling that stimulates their oxidation by the mitochondria to prevent their retention in the liver, thus promoting the production of oxidized and highly reactive species [39]. Furthermore, the occurrence of IR and the production of high levels of hormones (leptin, fibroblast growth factor-FGF), may lead to the desensitization of the organism to them or the progression of the steatosis and the promotion of fibrosis to the liver [40,41].

High levels of fats and sugars lead to the overactivation of the Krebs cycle, giving rise to highly reductive NADH_2_ and FADH_2_ and resulting in the production of intermediate oxygen-reduced products such as hydrogen peroxide and superoxide anion radical, which in the presence of free labile metals, due to the cellular damage from the NAFLD and especially NASH, also give rise to the highly reactive hydroxyl radical, with deleterious effects on mitochondrial membrane and DNA deterioration and deranged respiratory chain complex activity [42,43]. These effects, together with the increased influx of fatty acids and their induced lipotoxicity, create a toxic mixture rendering mitochondria dysfunctional, with further intensification of oxidative stress, ATP mal-production and cellular inflammasome activation and death [44,45]. In this direction, this also assists the FFA and their metabolic derivatives with gene-modulating activities [46]. Interestingly, in contrast to the lipotoxic effects of cholesterol and saturated fatty acids, the antioxidant mono-unsaturated fatty acids could reverse their induced cytotoxicity as antioxidant, anti-inflammatory and lipid-regulating moieties [47]. These actions of the latter may partly derive from their gene nuclear receptor ligand activation, such as the peroxisome proliferator-activated receptor-α (PPARα), with indirect modulatory effects on lipid oxidation, de novo fat synthesis, insulin sensitizing and decreased expression of inflammatory factors [like nuclear factor κB (NF-κB), TNF-α and IL-1β] [48,49]. In a similar manner, PPARγ directs the transition of the macrophages from the inflammatory (M1) to the anti-inflammatory (M2) phenotype. PPARδ attenuates the inflammatory responses of Kupffer cells and liver X receptors (LXRs) and adjusts the fatty acid synthesis and inflammatory processes, accentuating the additive role that some treatment options may have in NAFLD progression via gene modulation [50,51,52].

High levels of oxidized products and proteins accumulate in the ER, promoting ER stress, the activation of the unfolded protein response (UPR), and various downstream pathways, with activation of the protein kinase RNA-like ER kinase (PERK) and inositol-requiring signaling protein 1 (IRE1) that may interact with pro-apoptotic molecules like Bax and Bak or activate C-Jun N-terminal kinase (JNK) and give rise to apoptotic and decrease anti-apoptotic signals, causing cytochrome c release, apoptosis and inflammation, whilst JNK1 signaling suppression has been shown to abolish NASH development [53,54,55]. Additionally, oxidoreductin 1 alpha (Ero1α) and protein disulfide isomerase (PDI) are implicated in protein maturation in the ER, and their function may trigger the transfer of hydrogen atoms to molecular oxygen and the formation of ROS (mainly hydrogen peroxide), whilst disruption of these enzymes induces the misfolding of proteins and the imbalance between the disulfide and free thiol groups in the proteins, resulting in the production of more ROS [56]. As a result, the increase in misfolded and oxidized products in the ER gives rise to glutathione (GSH) consumption, with further oxidative imbalance [57]. Furthermore, UPR signaling could also downregulate nuclear factor erythroid 2–related factor 2 (Nrf2), which induces the expression of genes encoding or enhancing the expression of antioxidant elements such as heme oxygenase-1 (HO-1) and superoxide dismutase [58]. Thus, ER stress may even indirectly increase the oxidative insult in NAFLD since Nrf2 deletion has been shown to increase oxidative stress and promote the evolution of NASH [59].

In the interconnection between excess hepatic lipids and oxidation, another important factor is the effect of oxidative stress on adaptive stress responses, inducing typical redox-sensitive genes involved in the modulation of GSH levels and lipid metabolism [60]. Furthermore, nuclear receptors could serve as redox sensors modulating important processes as a response to ROS in the culture, with AMP-activated protein kinase (AMPK) regulation being one important example, with mitochondrial ROS inducing its activity, leading to regulation of a series of enzymes and receptors involved in lipid metabolism [such as acetyl-CoA carboxylase (ACC), PPARα and PPARγ] [31,61]. The AMPK activation has also to do with the increase in AMP/ATP ratio and the oxidation of its subunits from ROS and especially H_2_O_2_, which leads to the attraction of GSH and the glutathionylation of the protein, further promoting fatty acid β-oxidation [62]. However, the results are kind of ambivalent since hydrogen peroxide has been shown to downregulate PPARα expression and the expression of target genes that are needed for free fatty acid oxidation [63]. Furthermore, ROS and the lipid peroxidation final products, such as 4-hydroxynonenal (4-HNE), could also decrease the expression of PPAR-γ, which is implicated in insulin sensitivity, promoting insulin resistance and upregulating fatty acid synthetase and lipogenesis [64,65]. ROS-mediated insulin resistance could even be derived from the activation of kinases like JNK and p38 mitogen-activated protein kinase (MAPK) that catalyze the serine phosphorylation of insulin receptor substrate 1 (IRS-1), resulting in its inability to be activated by insulin via tyrosine residue phosphorylation and leading to its inactivation and degradation [66,67]. Thus, antioxidant compounds seem to alleviate hepatic steatosis via decrements in the expression of proteins implicated in de novo lipogenesis (such as sterol regulatory element binding protein 1c), implicating the role of ROS in lipid homeostasis and the positive effects of antioxidant treatments as direct radical scavengers, apart from their indirect modulatory effects [68].

Cumulatively, the pathogenesis of NAFLD is driven by a complex interplay between lipotoxicity, insulin resistance, and the liver–gut axis, constituting key mechanistic pillars that justify the exploration of multi-target therapeutic strategies, especially since these processes are closely linked to the overproduction of reactive oxygen species (ROS) and the activation of inflammatory pathways [69]. Insulin resistance plays a central role in amplifying these effects, as it promotes increased lipolysis in adipose tissue and enhances hepatic de novo lipogenesis, further exacerbating lipid accumulation in the liver [70]. The bidirectional relationship between insulin resistance and lipotoxicity creates a self-perpetuating cycle that accelerates disease progression. Equally important is the involvement of the liver–gut axis, which has emerged as a critical regulator in NAFLD pathophysiology. Alterations in gut microbiota composition and increased intestinal permeability facilitate the translocation of bacterial products such as lipopolysaccharides (LPS) into the portal circulation, triggering hepatic inflammation through toll-like receptor (TLR)-mediated pathways [71]. Furthermore, microbial metabolites, including short-chain fatty acids and bile acid derivatives, modulate host metabolism, immune responses, and insulin sensitivity, highlighting the systemic nature of these interrelated diseases [72].

Within this multifactorial framework, the use of natural compounds is increasingly justified due to their pleiotropic mechanisms of action. Many phytochemicals exhibit combined antioxidant, anti-inflammatory, insulin-sensitizing, and lipid-lowering properties, enabling them to simultaneously target multiple pathogenic pathways [73]. For instance, polyphenols such as resveratrol and curcumin have been shown to modulate NF-κB signaling, improve mitochondrial function, and influence gut microbiota composition, while compounds like chlorogenic acid and thymoquinone can attenuate oxidative stress and improve lipid metabolism [73,74,75]. This multi-target capacity contrasts with conventional monotherapies and supports their potential role as complementary therapeutic agents. Overall, a deeper understanding of the interconnected roles of lipotoxicity, insulin resistance, and the liver–gut axis underscores the need for integrated therapeutic approaches and provides a strong mechanistic rationale for the investigation of phytochemicals in NAFLD management.

## 2. Cinnamic Acid Derivatives

**Figure 3:**
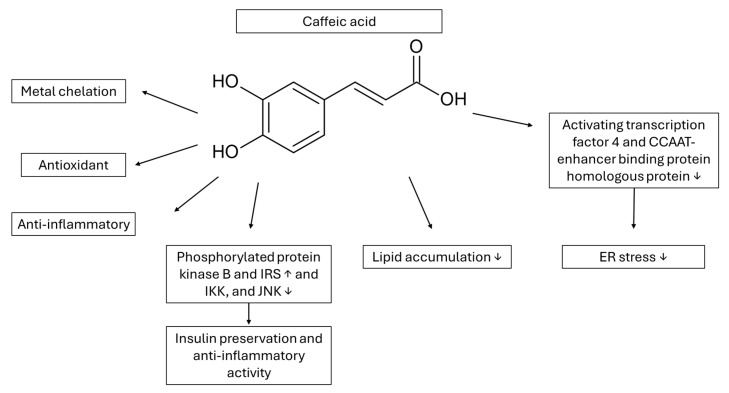
Properties of caffeic acid against NAFLD progression.

Among the cinnamic acid derivatives, caffeic acid (CA, 3′4′-dihydroxy cinnamic acid) and its derivatives have shown various antioxidant, direct and indirect properties, metal chelation and anti-inflammatory activities, with positive effects on obesity and serum lipid manipulation [76,77] (Figure 3). CA has been tested on palmitate-treated hepatic cell lines (AML12 hepatocytes, 50 μΜ CA concentration) and on mice fed a low-fat diet and obese mice fed a high-fat diet (HFD, 10 weeks, CA 50 mg/kg/day per os with their food) [78]. CA was not toxic to the cell lines up to 200 μΜ, and it decreased lipid accumulation up to 60% and the expression of genes concerning fatty acid synthesis and transport (fatty acid synthase, hormone-sensitive lipase, liver-type fatty acid binding protein, stearoyl-CoA desaturase1 and sterol regulatory element-binding transcription factor 1c) statistically significantly (*p* < 0.05), with half of these enzymes reaching the healthy group levels. Concerning the in vivo studies, CA decreased the HFD body weight and improved the glucose manipulation tested via glucose intraperitoneal administration, with serum insulin, triglycerides and total cholesterol levels decrease, improving both glycemic and lipid levels and hepatic steatosis. Additionally, its anti-inflammatory effects were also recorded at the cellular level with an antibody to phosphorylated protein kinase B and an increase in IRS-1 (insulin receptor substrate 1), and a decrease in IkB kinase (IKK) and JKK (c-Jun N-terminal kinase), with statistical improvement in ER stress and autophagy. Factors concerning ER stress (activating transcription factor 4, binding immunoglobulin protein, X-box binding protein 1, CCAAT-enhancer binding protein homologous protein) were significantly decreased, whilst autophagy markers (LC3, microtubule-associated protein 1 A/1 B-light chain 3; XBP-1, autophagy-related protein 7) were upregulated. These results were also verified in the HFD mice, with almost similar results taking place. Additionally, the normalization of ALT (alanine transaminase) and AST (aspartate transaminase) levels and the decrease in liver weight accentuate the protective role of CA on the liver and the accumulation of lipid droplets. Despite the promising results, the clinical application is limited. Caffeic acid has approximately 95% absorption from the human intestine, with the main problem after oral administration being the low oral bioavailability due to its solubility, which is 54% in gastric fluid (in vitro), combined with its fast metabolism due to its structure, which contains a lot of hydroxyls that favor a second phase of metabolism conjugation reactions [79]. Additionally, a study on CaCo-2 cell permeation showed low cellular permeation and 12% absorption in the rat’s intestine [79].

**Figure 4:**
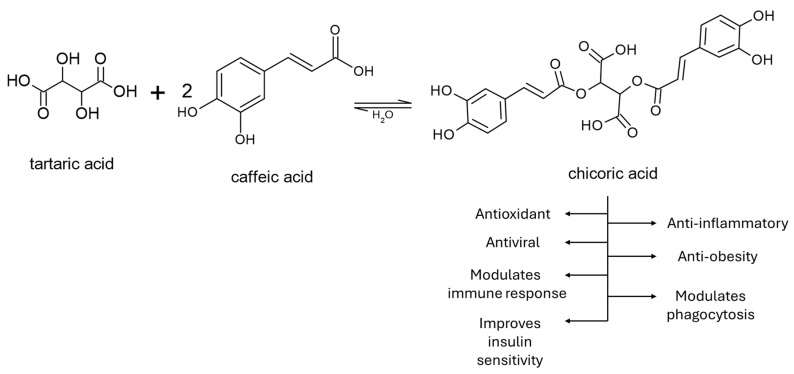
Structure, formation and pharmacological characteristics of chicoric acid.

Concerning CA, chicoric acid (CCA), a derivative of two caffeic acids esterified with tartaric acid and derived from *Crepidiastrum denticulatum*, possesses oxidation-inhibitory, fatty liver steatosis, diabetes type 2 and obesity modulatory activities [80,81] (Figure 4), whilst *C. denticulatum* extract (CDE) has also been shown to inhibit oxidatively driven liver damage [82]. CCA has been tested (10 or 30 mg/kg day) with CDE for comparison in mice fed a methionine- and choline-deficient (MCD, 25 or 100 mg/kg day) diet and in HepG2 and AML-12 cells [83]. Both CDE and CCA decreased in similar levels the steatosis, lobular inflammation and ballooning scores, with similar body and liver weight to the control group; however, CCA was at a much lower dose (30 instead of 100 mg per kg). Similarly, very substantial improvement was also recorded in the levels of ALT, AST, triglycerides and total cholesterol in blood, with parallel reduction of hepatic lipid accumulation, lipid peroxidation and fibrosis and reduction in fibrotic mRNAs and hydroxyproline content. Furthermore, induction of inflammatory and lipogenic genes was observed in MCD mice, which was attenuated in a dose-dependent manner by the extract and the compound itself, together with Nrf2 system induction, leading to NASH condition amelioration and apoptosis reduction by multiple interventions. Chicoric acid bioavailability is one limiting factor, which is approximately 2%, and its excretion has been up to 15% in rats’ urine [84]. The reason for these results is the poor lipophilicity, which extends to poor passive cellular permeability, resulting in low intestinal absorption.

**Figure 5:**
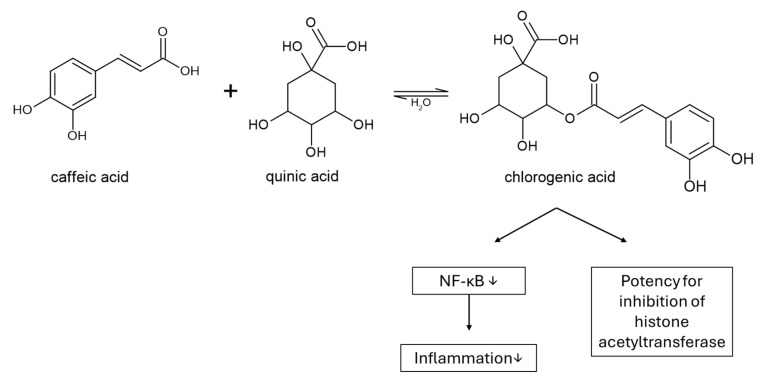
Origination and anti-NAFLD characteristics of chlorogenic acid.

Chlorogenic acid, a quinic acid ester (at 5 position) of caffeic acid, has been shown to possess histone acetyltransferase (HAT) inhibitory potency, with improvement in body weight, liver triglyceride (TG) levels, low-density lipoprotein (LDL), high-density lipoprotein (HDL), serum alanine aminotransferase (ALT) and serum aspartate aminotransferase (AST) levels, and a decrease in liver injury [85] (Figure 5). HAT enzyme is involved in both the first and second hits as a transcriptional coactivator [“two-hit” model of NAFLD progression]: the “first hit” is liver fat accumulation, which causes insulin resistance, and excess inflammation induced by inflammatory cytokine interactions acts as the “second hit” [86], and it works together with nuclear factor-κB to regulate various inflammatory signaling pathways. In bioavailability clinical studies, chlorogenic acid absorption in the small intestine seems to be one-third of the administered dosage. In the colon, a large amount reaches unchanged, where it is hydrolyzed by esterases and releases caffeic acid, which permeates cells through a passive diffusion mechanism [87,88].

Liver fibrosis is a common symptom of non-alcoholic steatohepatitis (NASH) and presents a significant global clinical challenge. It is a key pathological process in the progression of chronic liver disease to cirrhosis. The miR-122/HIF-1α signaling pathway is thought to play a critical role in the development of progressive fibrosis. Studies have shown that hepatic miR-122 levels are reduced in both patients and animal models with alcohol-related liver disease (ALD) or NASH, while hypoxia-inducible factor-1 (HIF-1), a target of miR-122, serves as a crucial regulator in the production of pro-fibrotic mediators [89,90].

Isochlorogenic acid B (ICAB), a compound naturally derived from Laggera alata (Asteraceae), has been used in traditional Chinese medicine for over three centuries, particularly for treating ailments associated with hepatitis [91,92]. To explore the effects of ICAB on liver fibrosis, a mouse model of NASH was induced using a methionine- and choline-deficient (MCD) diet for four weeks, with ICAB administered daily at three different doses (5, 10, and 20 mg/kg) [93]. Pathological assessments revealed that ICAB significantly alleviated liver fibrosis lesions. Treatment with ICAB (5, 10, and 20 mg/kg) resulted in a dose-dependent reduction in serum ALT and AST levels and greatly improved liver pathology, including hepatocyte ballooning and severe macrovesicular steatosis. Moreover, ICAB treatment reduced steatosis, as evidenced by significant, dose-dependent decreases in liver triglyceride (TG) and cholesterol (CHO) concentrations. ICAB also inhibited the activation of hepatic stellate cells (HSCs) and downregulated the expression of key genes involved in liver fibrosis, such as LOX, TGF-β1, MCP-1, COL1α1, and TIMP-1. Additionally, ICAB mitigated liver oxidative stress through the Nrf2 pathway and reduced fibrosis via the miR-122/HIF-1α pathway, indicating its potential as a therapeutic agent for liver fibrosis in NASH [93]. Thus, ICAB contributes to the improvement of NASH in preclinical evaluation studies through antioxidant, anti-inflammatory, and antifibrotic activity, similar to the approved medicine for NAFLD, Saroglitazar. The similarities are in the phenotype outcome, with differences in their molecular mechanisms. Saroglitazar achieves these effects by targeting the PPAR-α and PPAR-γ, focusing on the metabolic side [94], while ICAB acts on multiple pathways. Despite these promising results, ICAB’s bioavailability, which is similar to that of chlorogenic acid, may restrict its use as a potent treatment.

In a similar manner, two garlic skin-derived p-coumaric acid and ferulic acid (FA) derivatives, N-trans-coumaroyloctopamine (TCO) and N-trans-feruloyloctopamine (TFO), with various pharmacological properties [95] were tested in Sprague Dawley rats for their NASH activity via a high-sucrose and -fat diet (MCD) [96]. The antioxidants were administered 100 mg/kg body weight/day intraperitoneally for 8 weeks of diet. TCO derivative, a molecule with lesser antioxidant activity, could not offer a statistically significant reduction of both ALT and AST levels (only the ALT levels were decreased significantly), compared to TFO (derivative of the more active antioxidant ferulic acid). The latter could also decrease the MDA (malondialdehyde) and increase the SOD liver tissue levels. TFO offered normal liver histology, with a decrease in severe steatosis and inflammatory cell infiltration promoted via MCD and a decrease in the protein and mRNA levels of the inflammatory cyclooxygenase 2 (COX-2) in the hepatic tissue, reversing its expression to the levels of the healthy group. The liver-protective effects of TFO are in accordance with recent findings about ferulic acid [97]. In this study, FA (at different dosages of 25, 50 and 100 mg/kg for 4 weeks after 4 weeks of CCl_4_ administration) ameliorated the carbon tetrachloride-induced chronic inflammation and fibrosis, decreasing in a dose-dependent manner the ALT and AST levels and normalizing the oxidative and antioxidant marker levels (MDA, hydroxyproline and SOD). Furthermore, there was a decrease in fibronectin staining and fibrotic markers such as collagen 1, transforming growth factor- beta and Acta-2, with a parallel increase in phosphorylated AMPK levels and a decrease in the levels of ROS, without any sign of cellular toxicity at concentrations up to 100 μΜ. In the AMPK activation, PTP1Β (protein tyrosine phosphatase 1B) inhibition is involved, whilst AMPK inhibitors abrogated the anti-inflammatory and antifibrotic effects of FA. The above results indicate the dual anti-inflammatory and antioxidant hepatic cellular protective potential of the antioxidant cinnamic acid derivatives and their implication in the amelioration of liver fibrosis.

Rosmarinic acid is extracted from Rosmarinus officinalis and has many pharmacological properties, such as antioxidant, anticancer, antifibrotic, anti-inflammatory and anti-convulsant [98,99,100]. Its ability to inhibit the activation of nuclear factor erythroid-related factor 2 (NrF2) and heme oxygenase-1 (HO-1), seems to be related to the improvement it offers in CCl_4_-induced liver toxicity [101]. Additionally, in cellulo, in hepatic cells L02 treated with H_2_O_2_ and rosmarinic acid (in concentrations of 0.25 μM, 1 μM, and 5 μM), rosmarinic acid increased the viability of the cells and reduced ROS production [102]. Concerning the cell cycle and apoptosis, H_2_O_2_ increased the percentage of cells in phase G2, while the treated cells showed a reduction in phase G2 and G1 and an increase in phase S. Regarding the apoptosis, there was a decrease in the apoptotic cells but in a reverse-to-dose manner. The effect on oxidative stress was evaluated by the MAPK and Nrf2 pathways. The expression of Nrf2 and NQO1 was enhanced after the administration of rosmarinic acid, and the expression of JNK, p38 and ERK1/2 was decreased [102]. Rosmarinic acid, as a polyphenol, has low lipophilicity, rendering its bioavailability low. Studies in rats and humans have shown that the absorbed amount from the intestine is 1.69% of the orally administered dose [103,104]. Concerning its other pharmacokinetic characteristics, these are the same as those of chlorogenic acid. Except for caffeic acid metabolites, in humans and rats, conjugated forms of methylated rosmarinic acid, m-coumaric acid, ferulic acid and *m*-hydroxyphenylpropionic acid [105] have been found in urine. By this, we can hypothesize that part of the rosmarinic acid activity may be based on its metabolites, which may have better lipophilicity and bioavailability.

## 3. Tannins

**Figure 6:**
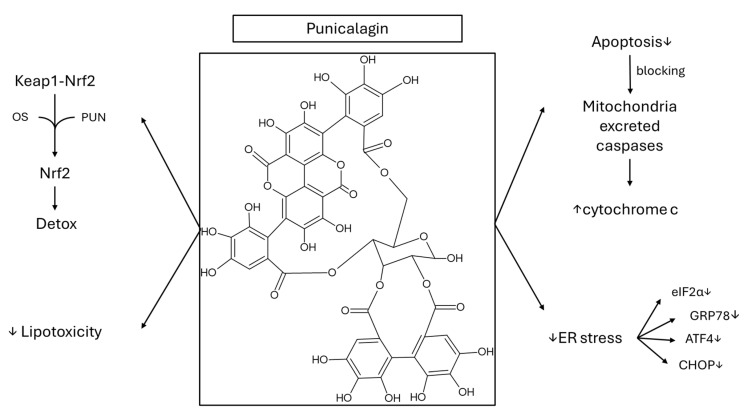
Structure and effects of Punicalagin.

Punicalagin (PUN), the most abundant ellagitannin in pomegranate, the fruit of Punica granatum, has been widely reported to be beneficial for various chronic diseases, activating the Keap1-Nrf2 antioxidant system (via phosphorylation of extracellular signal regulated kinase and improved nuclear translocation of Nrf2) in hepatic (HepG2) cells, attenuating palmitic acid (PA)-induced lipotoxicity and apoptosis via blockage of mitochondria-excreted caspases [106] (Figure 6). Additionally, PUN has shown to suppress ER stress liver damage [downregulated the expression of the markers eukaryotic initiation factor-2 alpha (eIF2α), glucose regulated protein 78 (GRP78), activating transcription factor 4 (ATF4) and C/EBP homology protein (CHOP)] in type 2 diabetes mellitus (T2DM)-induced mice, with insulin resistance, decrease in glucose and serum free fatty acids, insulin sensitivity and hepatic steatosis improvement in T2DM mice [107]. HFD rats treated with PE (PUN-enriched pomegranate extract, by oral gavage at 50 or 150 mg/kg/day) showed a statistically significant change in body weight at the high dose, with improvement in the serum parameters concerning lipids and diabetes mellitus [108]. The effects were dose-dependent with a decrease in insulin, HOMA-IR, TG, Cholesterol, LDL-C and leptin, and a decrease in HDL-C and adiponectin levels (in the cases of adiponectin and lipid markers, the levels were similar to the control). These effects were followed by improvement of serum ALT and liver weight, TG and cholesterol content, with normal H&E staining of liver tissue of animals treated with 150 mg of PUN. A decrease in the lipogenic proteins SREBP-1c, FAS (fatty acid synthase), ACC1 (acetyl-CoA carboxylase-1), and SCD1 (stearoyl-CoA desaturase-1) and in the lipid synthesis-related gene of ATP citrate lyase was shown, although there was an increase in diacylglycerol acyltransferases (DGAT1, DGAT2). In the same study [108], general anti-inflammatory status was shown with a decrease of up to 60% at 150 mg/kg for cytokines TNFα, IL-1β, IL-4, and IL-6, as well as immunoglobulins IgA, IgM, and IgG, whilst antioxidant liver potential was shown via a decrease in 4-hydroxynonenal (4-HNE) and improvement in SOD and GSSG (oxidized glutathione) levels. GSH levels were normal (decreased compared to the HFD group), potentially due to the increased antioxidant potential of PE, which offered a decrease in the oxidized GSH as well. These effects may also be related or lead (two-way interaction) to the improvement in mitochondrial function with ATP content increase and a decrease in uncoupling proteins (UCP2), accompanied by decreased protein carbonylation and increased activity of mitochondrial complexes I, II, and IV and expression of lipometabolizing proteins [peroxisomal proliferator-activated receptor–gamma coactivator-1α and -1β (PGC-1α and PGC-1β), PPAR-α, CPT1A and CPT1Β, acyl-CoA dehydrogenase of long chain, C2-C12 straight chain and very long chain (ACADL, ACADM, ACADVL)]. These effects may at least partly explain the decreased lipid content (TG, Cholesterol), with PUN showing similar effects on the markers, protecting against ROS production, and improving insulin signal transduction in the palmitate-induced HepG2 model. Since the PE extract contains PUN at almost 40% of its weight, PUN (50 mg/kg body weight/day by oral gavage) was also tested in a western diet (lower carbohydrates and proteins and increased lipids) for its ability to improve lipids and inflammation in the liver and the adipose tissue [109]. A decrease in liver enzymes and inflammatory cytokines was observed, with an increase in genes related to fatty oxidation and glucose intolerance and improvement in adiponectin levels and signaling, as well as lipid metabolism in adipose tissue. These effects were accompanied by enhanced gut barrier function and gut microbiome, improving the NAFLD-associated gut dysbiosis and ascertaining the multifactorial activity of PUΝ on NAFLD progression. PUN’s chemical structure indicates its high hydrophilicity, with low absorption and rapid metabolism. Thus, low concentrations end up in the systemic circulation, and this is a major limitation of that tannin despite its highly promising findings.

Other tannin-derived groups of compounds are schisandrins, found in the fruits of several species of the Schisandra genus, with antioxidant, cognitive improvement and drug-induced liver injury reversal effects [110,111]. The fully methylated Schisandrin A (SCHA, 0.05%, *w*/*w* of the diet) has been tested in male C57BL/6J mice fed with a high-fat and high-cholesterol (HFHC) diet [112]. Although SCHA did not alter the fat mass or the body weight, it significantly decreased FFA and triglycerides, with an increase in plasma HDL-cholesterol and a similar decrease in the hepatic FFA, TG and total cholesterol, increased β-oxidation (CPT1a, PPARα), and diminished lipogenesis [low FAS and phosphatidate phosphohydrolase (PAP)], but with a significant increase in the mRNA expression of genes controlling cholesterol homeostasis, including ABCA1 (member 1 of human transporter sub-family ABCA), SREBP2 and 3-hydroxy-3-methylglutaryl coenzyme A reductase. Furthermore, in the same study [112], a significant (*p* < 0.05) decrease in the hepatic thiobarbituric acid-reactive substances (TBARS, derived from reaction of thiobarbituric acid and MDA) and a very significant increase (*p* < 0.01) in the SOD, GPx and CAT levels was recorded. Analogous promising results for SCHA were shown in a very recent study of a high-fat diet [113]. In low (40 mg/kg) and high (80 mg/kg) doses, SCHA showed a decrease in body weight, with ALT, AST, liver MDA, SOD, TNF-α, IL-1β, IL-6, normalization, histological improvement, and a decrease in lipid accumulation, recorded by H&E staining and Oil Red O staining. Furthermore, an improvement in the metabolomics and steatosis profile was shown, with a decrease in oxidized 5-hydroxyindoleacetic acid, hydroxyproline, sphingoshine, oleamide and taurine ursodeoxycholic acid, and an increase in the amino acids and vitamins that are deteriorated during NAFLD progression. These effects were followed by glycogen synthase kinase-3 beta (GSK3β) inhibition, an enzyme involved directly or indirectly in adipogenesis through the upregulation of signal transducer and activator of transcription 5 (STAT5) and secreted frizzled-related protein (SFRP1) expression, reducing hepatic steatosis and oxidative stress-derived damage. In the same direction, Schisandrin B (SCHB), a cyclized derivative, has been shown to be implicated in several aspects concerning NAFLD, with a low dose for long-term treatment offering positive results compared to a high dose for short-term treatment that seems to be non-beneficial and, in some cases toxic [114]. At the protective levels, SCHB has been shown to inhibit adipogenesis (SREBP1, ACC and FAS expression) and TNF-α and IL-6 expression, with Nrf2 induction and ER stress amelioration, offering protection against toxic xenobiotics at the liver and inducing apoptosis in the HCC state [114,115,116].

Bicyclol (4,4′-dimethoxy-5,6,5′,6′-bis(methylene-dioxy)-2-hydroxymethyl-2′-methoxycarbonyl biphenyl) is a compound that is synthesized from Schizandrin C, a derivative of the plant *Schisandra chinensis*. Bicyclol has been used as a hepatoprotectant, while it exhibits antifibrotic properties and ameliorates liver damage in a dose-dependent manner in D-Galactosamine (GalN) and lipopolysaccharide (LPS)-induced liver failure models in mice [117,118,119,120]. Bicyclol also exhibits anti-inflammatory effects, as in mice treated with Concanavalin A, which induces liver injury. Bicyclol prevents the release of pro-inflammatory cytokines like TNF-α and IFN-γ [117]. In CpG DNa-stimulated L02 hepatocytes, bicyclol inhibits TLR9-mediated intracellular signaling pathways, including NF-κB and MAPK, significantly reducing the production of cytokines such as TNF-α, IL-18, MCP-1, MIP-1 and Rantes (C-C chemokine) [121]. Bicyclol’s antioxidant properties have been investigated through the analysis of mRNA levels of antioxidant enzymes. Bicyclol has been shown to induce the expression of glutathione-S transferase 1 (GSTA-1), heme oxygenase 1 (HO-1), and NADPH quinone oxidoreductase 1 (NQO-1), which are all enzymes implicated in the antioxidant defense of the hepatic cell, through the activation of the Nrf2 pathway [122]. In the same context, in mice treated with aflatoxin B1, bicyclol is associated with increased hepatic glutathione (GSH) levels and enhanced GSTA activity, providing strong protection against hepatotoxicity by neutralizing free radicals and reactive oxygen species (ROS) [123]. Regarding the antisteatotic effects of bicyclol, it reduces the accumulation of lipids in hepatic cells through the modulation of peroxisome proliferator-activated receptor α (PPARα), which regulates the β-oxidation of fatty acids. In mice, bicyclol administered three times a day for 2 days (75, 150, 300 mg/kg) also protected against tetracycline-induced fatty liver and reduced aminotransferase serum levels [124]. Thus, potentially this trifecta of crucial properties could establish bicyclol as a promising agent in ameliorating NAFLD.

Dimethyl dimethoxy biphenyl dicarboxylate (DDB) is another synthesized intermediate derivative of Schizandrin C (structurally similar to bicyclol), and it has been extensively used in the treatment of liver diseases, especially as an anti-hepatitis drug. DDB exhibits hepatoprotective, anti-inflammatory and antioxidant effects [125,126]. Dimethyl diphenyl bicarboxylate (DDB) has also shown its hepatoprotective efficacy across four liver injury models, CCl_4_-induced acute, DMN (dimethylnitrosamine)-induced subchronic, TAA (thioacetamide)-induced chronic and restraint stress-induced acute damage [127]. DDB significantly reduced serum AST and ALT levels and oxidative stress markers, while restoring key antioxidant enzymes in the CCl_4_ and DMN models. However, it showed limited efficacy in the TAA and restraint stress models. These findings suggest that DDB’s protective effects are model-dependent and highlight its potential therapeutic relevance primarily in chemical-induced liver injuries rather than stress-related hepatic damage. However, another rat model of high fructose-induced hepatic steatosis was employed to explore the effect of DDB on hepatic steatosis [128]. The rats were fed a high-fructose diet for 6 weeks, and then they were administered 300 mg/kg of DDB orally for an additional two weeks. DDB treatment effectively mitigated the adverse effects of a high-fructose diet, including significant reduction of liver weight, visceral fat, dyslipidemia, and insulin resistance. Moreover, it normalized serum ALT activity and lowered MDA levels. Histopathological analysis showed improved liver architecture, with minimal signs of swelling, degeneration, or steatosis, and necrosis being barely observed compared to the control group [128].

## 4. Miscellaneous Phenol-Containing Compounds

Gastrodin (GAS) is a substance extracted from *Gastrodia elata* and used in traditional Chinese medicine. Clinical uses of GAS on neurological disorders have been reported, while antioxidant and anti-inflammatory properties have also been observed [129,130,131]. The effects of GAS were studied in a high-cholesterol diet (HCD)-induced NAFLD larval zebrafish model [132]. Nile red staining was used to assess the lipid-regulating effects of GAS in HCD-induced larval zebrafish. The results revealed a dose-dependent reduction in red fluorescence intensity in the GAS-treated groups, indicating decreased lipid accumulation. Consistently, GAS significantly reduced triglyceride (TG) and total cholesterol (TC) levels in a dose-dependent manner. Furthermore, GAS improved oxidative stress markers, with all treatment groups showing a dose-dependent decrease in reactive oxygen species (ROS) and malondialdehyde (MDA) levels. At the molecular level, GAS attenuated the expression of lipogenesis-related genes, including sterol-regulatory element-binding proteins (SREBF1) and fatty acid synthase (FASN), while upregulating the expression of the lipid-lowering gene proliferator-activated receptor alpha (PPAR-α). Additionally, GAS mitigated inflammation, fibrosis, and oxidative stress by reducing the mRNA expression of pro-inflammatory cytokines, such as TNF-a, IL-6 and IL-1b, along with fibrosis-related genes. These findings suggest that GAS exerts protective effects against lipid dysregulation, oxidative stress, and inflammatory damage in HCD-induced zebrafish larvae. In another study, oleic acid (OA) was used to induce steatosis in cultured HL-7702 liver cells [133]. It was observed that GAS significantly reduced intracellular fat accumulation. Notably, GAS activated the AMP-activated protein kinase (AMPK) pathway, and its inhibitory effect on OA-induced fat accumulation was found to be AMPK-dependent [133]. In another study, OA was used to induce steatosis in HL-7702 cells, while a high-fat or high-fat/high-cholesterol diet was used to induce NAFLD in mice and rats [134]. GAS was dissolved in sterile saline and applied to cells at specific concentrations. After 24 h, total cellular proteins were collected for Western blot analysis. For the in vivo studies, mice were simultaneously treated with GAS at doses of 10 mg/kg, 20 mg/kg, or 50 mg/kg alongside a high-fat diet (HFD). GAS was dissolved in saline and administered orally to the mice once daily. The results demonstrated that GAS effectively mitigates oxidative stress and the proinflammatory response in OA-induced steatosis in cultured liver cells. GAS inhibited the OA-induced upregulation of proinflammatory cytokines, including TNF-α, IL-6, and cyclooxygenase-2 (COX2), while also reducing ROS and MDA levels. Notably, at a concentration of 100 μg/mL, GAS restored SOD, ROS, and MDA levels in OA-treated cells to near baseline values. In terms of lipid metabolism, GAS significantly decreased the mRNA expression of lipid synthesis-related genes, such as sterol regulatory element-binding protein 1c (SREBP1c), acetyl-CoA carboxylase 1 (ACC1), and fatty acid synthase (FAS). At the same time, it upregulated the expression of genes associated with lipolysis and fatty acid oxidation, including CPT1A and acyl-coenzyme A oxidase 1 (ACOX1). Additionally, GAS was found to activate Nrf2 and alleviate oxidative stress and the proinflammatory response via AMPK activation [134], indicating at the preclinical level a possible role for GAS in the treatment of NAFLD. Pioglitazone is an antidiabetic agent that is used for NAFLD treatment, as it can improve IR, enhance PPAR activity and treat type 2 diabetes, a trigger point of this disease [135]. Gastrodin has effects in common with pioglitazone, which is beneficial for NAFLD treatment; both reduce lipid accumulation, oxidative stress and inflammation. However, they differ in their mechanisms: Gastrodin modulates AMPK and NRf2 pathways, enhancing fatty acid oxidation and antioxidant defense. Pioglitazone acts as a PPAR agonist and, at the same time, improves insulin sensitivity. Including these findings and gastrodin’s good bioavailability, with the absorption in in vivo studies being more than 80%, and alongside the rest of its very promising pharmacological profile, gastrodin may be a good supplementary addition in the management of NAFLD [136]. However, clinical studies must be performed to confirm its applicability.

Salidroside is a secondary metabolite of the herbal medicine Rhodiola Rosea L. It is a phenolic compound with cardiovascular and central nervous system activity, and anti-hypoxic, anti-inflammatory, anti-aging and antioxidant properties [137]. A high-fat and high-cholesterol (HFHC) diet was used on male Sprague Dawley rats to provoke the NAFLD conditions. The NAFLD groups were fed with an HFHC diet for 14 weeks (the administration of salidroside was intragastric, at a dose of 150 mg/kg and 300 mg/kg, started from the ninth week) [138]. The liver enzymes ALT, AST and serum TC and TG were reduced dose-dependently in the treatment groups compared to the untreated group. The antioxidant properties were evaluated by the oxidative stress marker (SOD, GSH, GPX, CAT and MDA) changes. The activity of the antioxidant enzymes in the treated groups increased by up to 50% at the highest dose, while the MDA levels were decreased compared to the NAFLD group. Additionally, a reduction in mRNA expression of the radical-producing cytochrome (CYP) 1A2 was recorded, which is a major producer of ROS in the liver [139]. In addition, the gene expression of Nox2 decreased in a dose-dependent manner. This marker indicates an anti-inflammatory action of salidroside, as the NO is part of inflammation activation and peroxynitrite formation. The limiting factor of this molecule is the variation in bioavailability. The maximum bioavailability in Caco2 cells was 2.68%, while in gastrointestinal fluid it was 98%, and in rat plasma after oral and intravenous administration it was 32–98% (with a main factor for this variation being the differences in the administered dose) [140].

Protocatechuic acid is a metabolite of anthocyanins, a water-soluble subgroup of flavonoids, primarily found in Salvia miltiorrhiza and Hibiscus sabdariffa [141]. It possesses antioxidant and antihyperlipidemic properties [142]. It has been shown to enhance insulin pathways, thereby improving insulin uptake by the liver [143]. Studies in diabetic rats indicated that protocatechuic acid improves vascular activity [144]. In this study [144], dexamethasone was used to induce NAFLD conditions, as it causes insulin resistance, promotes lipogenesis, and elevates TG levels in the liver. The researchers employed Wistar rats that were administered protocatechuic acid orally for 14 days at doses of 50 mg/kg or 100 mg/kg, with dexamethasone administration beginning on the seventh day of treatment via intraperitoneal injection (1 mg/kg). Body weight was significantly decreased only in the high-dose group, whilst fasting glucose and insulin levels decreased in a dose-dependent manner (as did HOMA-IR). The phosphorylation of AKT at ser473 was not enhanced in the low-dose group. Both treated groups reversed vascular relaxation by increasing acetylcholine levels in a dose-dependent manner. Serum TC, TG, LDL and VLDL levels decreased, while HDL levels increased in a dose-dependent manner, with similar results being observed for the hepatic enzymes ALT and AST. Regarding antioxidant activity, only the high-dose group showed a reduction in MDA levels, while SOD activity increased in both groups. Additionally, in a dose-dependent manner, the administration of protocatechuic acid successfully elevates eNOS mRNA expression and reduces NOX4 levels, and in the same manner, the mRNA expression of enzymes phosphoenolpyruvate carboxykinase (PEPCK) and glucose-6-phosphatase (G6Pase), which are related to gluconeogenesis and blood insulin levels, respectively (they were decreased after one week of protocatechuic acid treatment).

**Figure 7:**
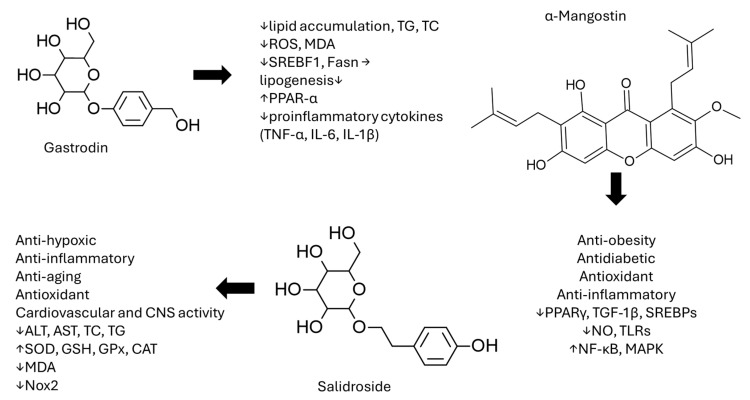
The effects of gastrodin, salidroside and α-mangostin on NAFLD.

Belonging to the category of xanthonoids, α-mangostin (AMN), obtained from the bark and dried sap of Garcinia mangostana L., has been shown to bear anti-obesity, antidiabetic, antioxidant and anti-inflammatory characteristics, with a reduction in body weight and adipose tissue and an increase in fatty acid oxidation [145,146] (Figure 7). Its wide array of effects are related to various mechanisms, including hepatic AMP-activated protein kinase and Sirtuin-1 activation, and reduction of peroxisome proliferator-activated receptor γ expression, transforming growth factor-β1 (TGF-1β) pathways and the sterol regulatory-element binding protein, with parallel modulation of nuclear factor-κB and mitogen-activated protein kinase activation and reduced expression of inducible nitric oxide synthase toll-like receptor [146]. As for NAFLD specifically, AMN in high-fat diet-induced obese mice significantly reduced body, liver and fat weight, with glucose, triglycerides and insulin-modulating effects in the plasma [147]. In the same study, adiponectin was increased, with a decrease in the expression of macrophage markers and inflammatory cytokine genes (TNFα, IL-1β, IL-6, and MCP1), attenuating infiltration of macrophages in white adipose and liver tissues and suppressing the migratory ability of immune cells and the mediators of fibrosis. Besides these positive findings, the lack of clinical trials alongside the low bioavailability and rapid metabolism of AMN are the limiting factors in order to be proposed as a treatment. However, in rats, the dilution of AMN in oil led to very good distribution in all tissues [148,149].

The combination of naturally occurring phenolic antioxidants, such as Garcinol, a polyisoprenylated benzophenone extracted from *Garcinia indica*, and curcuminoids, namely curcumin, demethoxycurcumin and bisdemethoxycurcumin, which comprise the Curcumin C3 Complex, was also evaluated as a potential supplement in the treatment of NASH and prevention of disease progression. Garcinol has been shown to have a protective effect against drug-induced hepatotoxicity and fibrosis, as well as anticancer action against leukemia cell lines [150,151,152]. Curcuminoids have also been associated with antioxidant and hepatoprotective ability [153]. Evidence demonstrates that curcuminoids exert hepatoprotective effects against CCl_4_-induced toxicity by mitigating fibrosis and inflammation [154]. Furthermore, their protective mechanisms against liver damage are associated with a reduction in oxidative stress and the suppression of nuclear factor kappa B (NF-κB)-mediated inflammatory pathways, as demonstrated in NASH-HCC mouse models [153,155]. A study evaluating the combined effects of garcinol and curcuminoids against NASH was applied using a Stelic animal model (STAM) of NASH; the Garcinia indica extract (GIE) was standardized to contain 20% *w*/*w* garcinol and 95% *w*/*w* curcuminoids derived from Curcuma longa [156]. The tested substances or vehicle were administered orally to mice at a volume of 5 mL/kg body weight once daily for four weeks. The NAFLD activity score (NAS) was statistically significantly lower in the combination treatment group, while inflammation also significantly decreased. Liver fibrosis was decreased approximately by 30%, the mRNA expression of collagen 1 and TGF-β, which are indicative of fibrosis, were significantly lower and liver hydroxyproline concentrations were also lower. CRP mRNA levels and both mRNA and protein levels of TNF-α were decreased in combination-treated mice livers, showcasing the anti-inflammatory effect of the combination [156]. These findings are in accordance with clinical evidence of curcumin, which showed a significant decrease in the ALT and AST levels of NAFLD patients, whilst longer duration of supplementation also resulted in a decrease in ALP [157]. Additionally, curcumin has shown synergistic effects at the in vivo level when co-administered with saroglitazar (a PPARα/γ agonist tested for NAFLD treatment), compared to curcumin mono-treatment, indicating the co-treatment efficacy of these compounds, especially as supplementary treatment options [158]. However, the lack of clinical translation studies of the combination of garcinol and curcuminoids, and the low bioavailability of curcuminoids and garcinol, accentuate the need for further studies [159,160].

## 5. Stilbene Derivatives

Among the stilbene derivatives, the most tested for liver toxicity and NAFLD treatment is resveratrol (RSV). Resveratrol is a polyphenol isolated from plants such as grapes, peanuts, and Polygonum cuspidatum [161]. It is a phytoestrogen with estrogenic properties [162] that can decrease oxidative stress and inflammation by activating the cAMP-PRKA-AMPK-SIRT1 pathway, also known as silent information regulator 2 homolog 1. This pathway also activates the autophagy of cancer cells [163]. In addition, it can regulate lipid metabolism [164].

Resveratrol has been tested (administered orally at 80 mg/kg) on piglets, with NAFLD conditions provoked by lower body weight due to intrauterine growth retardation (IUGR), which is associated with the disease [165]. The results for the treated group showed that TG and FFA levels were significantly reduced, while liver LPL and serum ALT levels were decreased, and AST levels remained unaffected. The biomarkers of oxidative stress, MDA and protein carbonyls were reduced, with the activity of glutathione reductase and glutathione being increased. The inflammation markers TNF-α and monocyte chemotactic protein 1 (MCP1) were reduced; however, NF-κB, IL6, and IL10 remained unaffected. Resveratrol has also been shown to act against non-alcoholic steatohepatitis by activating the AMPK/Nrf2 signaling axis, alleviating liver injury from a high-fructose diet by enhancing antioxidant defenses and modulating lipid metabolism [166]. The effectiveness of resveratrol in hepatic fat and ALT level reduction is comparable to resmetirom, highlighting these findings as a comprehensive protective effect against oxidative stress and lipogenesis in the liver; however, the low oral bioavailability, rapid metabolism and fast excretion of resveratrol render its applicability arduous [167,168].

Lower levels of estrogens are associated with metabolic syndrome, as well as night work compared to day work. A relevant study evaluates the efficacy of the combined treatment of resveratrol and melatonin [169], a regulator of the circadian cycle that has been shown to reduce hyperglycemia, hyperinsulinemia, total cholesterol (TC), and triglycerides (TG) [170]. The researchers used female Sprague Dawley rats, which were exposed to 12 h of light and 12 h of darkness. In addition, half of the rats underwent ovariectomy, and the conditions for non-alcoholic fatty liver disease (NAFLD) were induced by a 30% fructose diet in their drinking water [169]. The combined treatment reduced body weight, BMI, intra-abdominal fat, and the adiposity index by 16%, 19%, 36%, and 26%, respectively. Furthermore, the reduction in serum glucose, cholesterol, triglycerides, and free fatty acids (FFAs) was more efficient than the monotherapies.

The intracellular cyclic adenosine monophosphate (cAMP) activates AMP-activated protein kinase (AMPK) and subsequently SIRT1, a regulator of autophagy, necessary for the beneficial effects of resveratrol [171]. The cAMP-PRKA-AMPK-SIRT1 pathway, which activates autophagy, may enhance the antioxidant activity of resveratrol to alleviate NAFLD. A study was conducted on 129/SvJ mice fed a high-fat diet for four weeks, followed by an additional four weeks with resveratrol added to the diet [172]. Firstly, resveratrol decreased lipid accumulation in hepatic cells induced by palmitic acid and increased the expression of LC3-II, a protein related to the autophagy of neurodegenerative diseases, while reducing SQSTM1, a protein that synthesizes p62 linked to the autophagosome membrane. The expression of SIRT1 was increased by resveratrol both in vivo and in vitro, with resveratrol also inhibiting the activity of the AMPK inhibitor CC and decreasing the levels of the PRKA inhibitor H-89. Finally, serum TG levels decreased in a dose-dependent manner.

Praziquantel is a drug used to treat helminthiases. Its side effects also concern the liver [173]. Thus, it is combined with antioxidants or hepatoprotective factors. In a study, 10-(6′-plastoquinonyl) decyltriphenylphosphonium (SkQ1) (as a mitochondrial antioxidant) and resveratrol were used [174,175]. A study used Syrian hamsters infected with Opisthorchis felineus, which is known to cause cancer of the bile duct. They tested praziquantel (400 mg/Kg) alone and in combination with SkQ1 (1 μM/Kg) and resveratrol at doses of 1 mg/kg and 50 mg/kg [175]. A significant reduction in serum ALT levels was observed only in the group that received resveratrol. All treatment options reduced bile duct proliferation, inflammation, periductal fibrosis, and cholangiocyte hyperplasia. Compared to praziquantel monotherapy, lipids were not found scattered with the combined treatments involving the antioxidants. Similar results were noted for glycogen accumulation. Fibrosis was assessed by evaluating the gene expressions related to Alox5, Acta2, Tnfa, and Ck7, which did not show significant effects. However, the impact of oxidative stress was positive, measuring the reduction in 4-hydroxynonenal with the combined treatment, accentuating the ambivalence of findings and the need for further research.

Resveratrol has been found to modulate gut microbiota by decreasing Enterococcus faecalis and increasing Lactobacillus and Bifidobacterium. NAFLD is associated with increased gut permeability and reduced expression of tight junction proteins [176]. Thus, this study was designed to check the interaction between resveratrol and gut microbiota and its contribution to the amelioration of NAFLD [176]. The researchers used C57BL/6J mice that were fed a high-fat diet to achieve the NAFLD conditions, and resveratrol was administered by oral gavage at 300 mg/kg [177]. Resveratrol decreased body weight, TG and TC, as well as oxidative stress, as the decrease in MDA level and GSH-Px and SOD activity restoration showed. The mRNA expression of intestinal physicochemical barrier proteins, such as goblet cell protein TFF3, the major mucin genes (Muc1 and Muc2), resistin-like molecule beta (Relmβ) and regenerating islet-derived protein-3γ (Reg3γ), was restored in the treatment group compared to the untreated group that were downregulated. Finally, in the treatment group, there was diversity in the gut microbiome compared to the diseased group. This phenomenon might depend on insulin resistance, which was improved, together with inflammation. In another study, a high-fat diet was applied to induce NAFLD conditions in Wistar rats, and resveratrol (400 mg) was added to the diet from the start or one week later [178]. The efficacy comparison was conducted on male and female rats. In both female and male rats, ALT levels were reduced, while triglyceride levels remained stable. The expression of inflammation-related genes, such as COL1α1, TGF-β1, TNF-α, UCP2, SREBP-1c, and PGC-1α, also remained stable, indicating that the early introduction of resveratrol could alleviate the histopathology of NASH. In another study, rats were given a high-fat, carbohydrate-free diet supplemented with resveratrol for four weeks. The results showed a significant reduction in hepatic steatosis, which was attributed to the suppression of TNF-α secretion [179]. Resveratrol demonstrated hepatoprotective effects by reducing hyperlipidemia and steatohepatitis. Evidence for this comes from a study where mice with steatosis induced by an atherogenic diet were supplemented with resveratrol for eight weeks [180]. Similarly, in a high-fat diet-induced obesity and fatty liver model using C57BL/6J mice, resveratrol supplementation partially prevented steatohepatitis and hepatic ballooning [181].

At the clinical stage, in a double-blind clinical trial, patients with NAFLD (verified by a biopsy) were given resveratrol 500 mg, three times per day, or placebo for three years [182]. The results showed that ALT, AST, and GGT (gamma-glutamyl transferase) levels reduced by up to 25% by the end of the treatment in the resveratrol group, whilst the metabolic syndrome markers, such as BMI, body weight, and waist circumference, remained unchanged. Another double-blind clinical trial also used 500 mg of resveratrol compared with a placebo once daily for 12 weeks [183]. A higher reduction was observed in the serum levels of ALT and AST in the treatment group (compared to the control). The inflammation markers, such as high-sensitivity C-Reactive Protein (hs-CRP), IL-6, and NF-kB, were significantly reduced (*p* < 0.05) in the resveratrol group. Finally, the hepatocellular apoptosis biomarker cytokeratin-18 M30 decreased significantly compared to the placebo group. However, ambivalent findings are recorded for resveratrol, with a double-blind clinical trial involving males with NAFLD having found that taking 1500 mg of resveratrol in the morning and at night for 8 weeks did not benefit patients [184]. Resveratrol did not improve insulin resistance nor the transcription of NQO1, PTP1B (Protein Tyrosine Phosphatase 1B), IL-6, and HO-1 genes. In addition, the hepatic markers ALT, AST, HDL, LDL, and TG did not change, whilst regarding inflammation and oxidative stress, TNF-α, SOD, GPx, and TAC (total antioxidant capacity) did not change either. These results accentuate the need for more multi-centered and long-term studies for the verification of the efficacy of resveratrol, also showing the differences between the clinical studies, or the in vivo and clinical findings that stand as main obstacles towards the progression of the evaluation of resveratrol against NAFLD.

**Figure 8:**
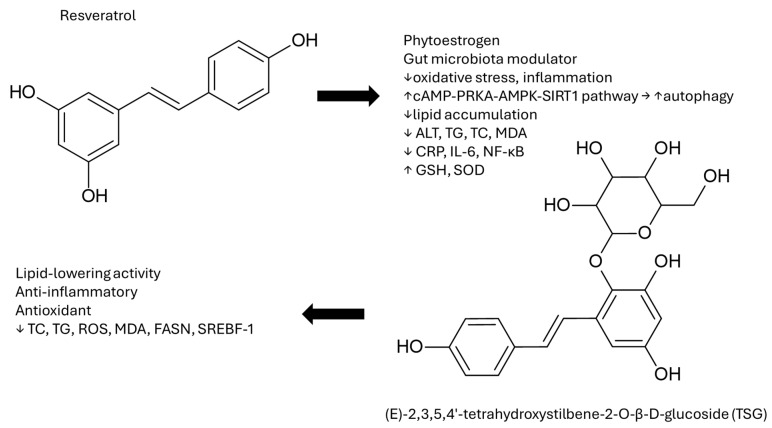
Structure and effects of resveratrol and TSG.

Structurally similar to resveratrol is (E)-2,3,5,4′-tetrahydroxystilbene-2-*O*-β-d-glucoside (TSG), derived from Polygonum multiflorum Thunb, which expresses lipid-lowering and anti-inflammatory activities, together with its antioxidant effect [185,186]. The effects of TSG (dose 25, 50 and 100 μg/mL of cultured zebrafish water) on larval zebrafish for 10 days were tested with hepatic steatosis via a high-cholesterol diet [187]. TSG supplementation resulted in an increase in the survival and body mass index, and a decrease in the weight and the width of larvals, with all the recorded results being dose-dependent in all the experiments. Liver and microvascular steatosis were decreased up to 75% at the high dose, with cholesterol and triglyceride levels being comparable to the control group, resulting in very low cholesterol deposition in the liver, as the Oil Red O staining dictated. The results were similar for ROS production, MDA and non-esterified fatty acid levels, with statistically significant increases (*p* < 0.01 for low doses and *p* < 0.001 for high doses) in the levels of SOD (superoxide dismutase) activity, the ratio of SOD/MDA and the mRNA expression of PPARα and PPARγ compared to the non-treated group. Furthermore, TSG decreased the level of expression of the fatty acid synthase (fasn), the sterol regulatory element binding transcription factor 1 (srebf1) and of the carnitine palmitoyltransferase 1a (cpt1a), signaling the fact that it can also be implicated in lipogenesis and lipid transfer to the liver, accentuating in these preliminary results its potency against many of the “multiple hits” involved in NAFLD progression (Figure 8). On the other hand, its poor stability, poor absorption in the intestine, and short-term storage in tissues are some of its drawbacks, showing, at least partly, poor pharmacokinetic behavior [188].

## 6. Coumarinoids

Scoparone (SCOP, 6,7-dimethoxycoumarin, 6,7-dimethylesculetin) is the major component of Artemisia capillaris Thunb., with effects on hepatic and cholestatic disorders, including antifibrotic, antioxidant, antiapoptotic and anti-glycemic effects [189,190]. SCOP inhibits adipogenesis (with reduction in triglycerides), mediated by peroxisome proliferator-activated receptor (PPAR) γ and CCAAT/enhancer binding protein (C/EBP) transcription regulation, resulting in a decrease in lipid droplet formation, together with decreases in lipid biosynthesis and gluconeogenesis critical proteins like sterol-regulatory element binding protein 1c (SREBP1c) and fatty acid synthase (FASN) [191,192]. Coumarin itself (30 mg/kg) and coumarin derivatives—esculetin, scoparone and 4-methylumbelliferone (all tested at 35 mg/kg)—have been tested on acute CCl_4_-induced toxicity for their hypolipidemic potency, with all the coumarins offering a statistically significant decrease in some of the lipidemic markers (cholesterol, triglycerides, VLDLC-very low density cholesterol and HDLC-high density lipoprotein cholesterol), but only esculetin and scoparone offered significant improvement in all the markers [193]. Liu and his colleagues, in two studies, have shown to alleviate NASH progression in MCD (for 4 weeks)-treated mice, with SCO used at 20, 30 and 80 mg/kg intraperitoneally daily [194,195]. The effects of SCO, in almost all cases, were dose-dependent, with a decrease in body weight and a decreased ratio of liver to body weight. Decreases in hepatocyte ballooning, steatosis and lobular inflammation scores were recorded, with ALT, AST and triglyceride levels decreasing up to normal levels in parallel with the apoptosis index and the caspase-3 protein expression levels, also followed by a decrement in inflammatory cytokine mRNA levels, like IL-1β, TNF-α, and collagen fiber and lipid droplet staining, and an increase in the anti-inflammatory IL-10. These effects were in accordance with the observed macrophage cell proliferation and cytokine generation inhibition induced by serum lipopolysaccharide (LPS) administration in RAW264.7 mouse and AML12 cells, and the TLR4 (toll like receptor 4)/NF-κB, ROS/P38/Nrf2 and PI3K/AKT/mTOR signaling pathway blockage observed in these studies, enhancing autophagic flux (since chloroquine, an autophagic flux inhibitor, significantly inhibited the anti-inflammatory effect of SCO) in macrophages but not in hepatocytes. Despite the mechanistically positive effects of SCO in preliminary studies, its scant toxicity data and its rapid metabolism (as some data from various strains of animals show) may question its applicability and accentuate the urgent need for clinical evaluation [190].

Concerning 4-hydroxycoumarin (umbeliferone, UMB), it has shown its activity against inflammatory, oxidative and metabolic disorders, with significant antitumor activity [196,197]. UMB was tested (30 mg/kg by gastric gavage) on HFD male C57BL/6J mice (12 weeks) and on AML12 cells treated with PA [198]. UMB showed significant changes in body and liver weight compared to the non-treated group, with a decrease in serum levels of ALT, AST, glucose, insulin and lipidemic indices (TC and TG), with the decrease in liver levels being similar (and not statistically different) to those of the regular-diet mice. As an extent, UMB improved the glucose tolerance test (GTT) and the insulin tolerance test (ITT), indicating the regulation by UMB of the increased insulin secretion during the HFD. Furthermore, UMB decreased lipid droplets and the expression of lipogenesis-related proteins (SREBP1, FAS, ADRP, and PPARγ) together with ROS, apoptosis and ER stress promotion (improvement in the levels of activating transcription factor 4, binding immunoglobulin protein, C/EBP homologous protein, SOD, HO-1, BCL-2 associated X-protein, B-cell lymphoma 2 protein and cleaved caspase-3). UMB has also shown to improve the diethyl nitrosamine (DEN)-induced hepatocellular carcinoma, offering antioxidant, pro-inflammatory cytokines and mitochondrial dysfunction inhibitory activity [199]. UMB was used as umbelliferone β-D-galactopyranoside isolated from plants and was loaded in poly(d,l-lactide-co-glycolide) nanoparticles for improvement of its pharmacokinetic characteristics. These results dictate the potential of UMB to reverse NAFLD and the derived HCC, whilst application of nanoformulations of UMB may be required since bioavailability issues may arise, as is the case with most of the lipid-soluble coumarinoids [200].

Wedelolactone is a coumarin derivative with many pharmacological activities, such as antifibrotic effects on human stellate cell line LX-2, inhibitory effects on proliferation and differentiation of pre-osteoclasts and anti-HCV activity [201,202]. The compound has been tested (400 mg/Kg) on acute hyperlipidemia, induced in KM mice by Triton WR-1339 (tyloxapol), and in hyperlipidemia induced by a high-fat diet on male Syrian golden hamsters [203]. In both tests, the administration of wedelolactone was intragastric (in the first study at a dose of 100 mg/kg, and at doses of 10, 25, and 40 mg/kg in the second study). The results of the high-fat diet showed that the activity of wedelolactone can modulate, in a dose-dependent manner, PPARα, AMPK, LDL receptor and LPL expression. All these factors contribute to lipid modulation. Also, the decrease in serum TG, LDL and ALT levels was evident; however, it was statistically significant only at 25 and 40 mg/Kg. However, in evaluating the liver TC and TG, the decrease was similar at the three doses for both factors, and it was up to 35% and 22%, respectively, compared to the NAFLD group. In the same study, the antioxidant effect of webelactone in vitro and in vivo was assessed [203]. In vitro, in the DPPH experiment, it reached an IC_50_ of 46 μg/mL, comparable to trolox, a highly antioxidant molecule. In vivo, the activity of SOD and GSH-Px and the formation of MDA were tested. SOD activity in the treatment groups was similar to that of the control group and higher than that of the untreated group. GSH-Px activity was also higher than in the diseased group but not as high as the activity of the control group, with an explanation for this possibly being the association between PPARα expression and SOD activity on hepatic cells, since wedelolactone has the ability to activate PPARα and AMPK, which are associated with the catabolism of lipids and the combined antioxidant activity.

## 7. Quinone Derivatives

Acetylshikonin (AS) is a naphthoquinone compound derived from Zicao, a traditional herbal medicine, with wound healing, antidiabetic and anti-inflammatory characteristics, together with lipid accumulation inhibitory potency and lipolytic activities [204]. Additionally, it has also been shown to prevent obesity (54.4% decrease) in genetically diabetic–obese (db/db) mice (AS treatment 540 mg/kg/day by oral gavage), with a decrease in the food efficiency ratio and no pathological signs in the tested animals [205]. Furthermore, body fat, adipose tissue and body mass index (BMI) reduction was observed up to 17.1%, with significant inhibition of deposition of lipid droplets in the hepatocytes and a decrease in plasma lipidemic indices (triglycerides, free fatty acids), glucose, and AST and ALT liver enzymes. Furthermore, a statistically significant (*p* < 0.05) increase in the expression of lipid metabolizing enzymes [perilipin, hormone-sensitive lipase (HSL), adipose TG lipase (ATGL)] and a decrease in the expression of lipid synthesis-related proteins [sterol regulatory element-binding protein-1 (SREBP-1), fatty acid synthetase (FAS) and 3-hydroxy-3-methylglutaryl-coenzyme A reductase (HMGCR)] was shown, with the ability to reverse the increase in the levels of pro-inflammatory cytokines (TNF-α, IL-6, IL-1β), accentuating the positive relation of AS with a wide array of mechanisms implicated in NAFLD. AS has also been tested directly for NASH treatment in a methionine–choline-deficient (MCD) diet in mice at doses of 270, 540 and 1080 mg/kg/day intragastrically daily for 5 days [206]. AS alleviated hepatic steatosis and inflammation, with a decrease in inflammatory cell infiltration, ballooning degeneration and lipid deposition in the liver, dose dependently. Additionally, hepatic levels of triglycerides were decreased, with autophagy (increased formation of autophagosomes) involvement in the process, but with a significant decrease in IL-1b and TNF-a and apoptosis of hepatocytes. AS evidently decreased the expression of fibrotic indicators such as a-SMA, collagen I, collagen III and fibronectin, and it dramatically decreased the levels of phosphorylated mammalian target of rapamycin (mTOR) and increased those of AMP-activated protein kinase (AMPK) phosphorylation, implicating the potential involvement of the AMPK/mTOR pathway in AS-induced autophagy. These results confirm the ameliorating effects of AS in NASH, with autophagy induction at least partly being one of the reasons, since chloroquine administration with AS notably counteracted its beneficial effects.

Concerning quinone derivatives, cryptotanshinone (CT), an abietane diterpene derived from the root of Salvia miltiorrhiza (a potent antioxidant and lipid peroxidation category of inhibitors with effect on metabolic disturbances [207,208]), has been tested (at 20 and 40 mg/kg orally) for its hepatoprotective activity in ethanol-induced liver injury in C57BL/6 mice [209]. The effects were dose-dependent, with normalization of the liver triglycerides and the ratio of liver to body weight, and with improvement in liver morphology and a decrease in fat deposits (depicted with hematoxylin and eosin staining) and lipid droplets (with Oil Red O staining). Furthermore, no cytotoxicity was observed in cells treated with CT (HepG2 and AML12 cells), whilst TG accumulation inhibition of almost 40% was observed in AML12 cells. Additionally, normalization (at 40 mg of CT) was observed of the lipogenesis proteins mRNA [sterol regulatory element-binding protein-1c (SREBP-1c), fatty acid synthase (FAS) and stearoyl-CoA desaturase-1 (SCD1)], with stimulation of fatty acid oxidation, as the relative mRNA of peroxisome proliferator-activated receptor α (PPARα), carnitine palmitoyltransferase-1α (CPT1), and acyl-coenzyme A oxidase (ACO) showed. These results seem to be related, partly, to AMPK/SIRT1 pathway activation and the observed modulation of the SOD, GPx, CAT, TBARS and CYPR2E1 mRNA levels that lead to a decrease in the oxidative stress and the induction of hepatotoxicity, with a very statistically significant improvement in the inflammatory state [decrease in TNF-α, IL-6 and MCP-1 (monocyte chemotactic protein-1)]. These results, although they do not concern NAFLD amelioration, offer ample evidence about the hepatoprotective, antisteatotic and antioxidant effects of CT, rendering it as a potential tool whose action remains to be elucidated and verified. However, another similar derivative, Tanshinone IIA, the unsaturated furane derivative of CT, has similarly been shown to act as a modulator of IL-1β, IL-6, TNF-α, the high mobility group box 1 protein (HMGB1)-Toll-like receptor 4 (TLR4)/NF-κB pathway and the activation of phosphatase and tensin homolog deleted on chromosome ten (PTEN)/phosphatidylinositol 3 kinase (PI3K)/AKT pathway, improving fatty liver conditions and lipid peroxidation processes (regulating PPAR-α, MMP2 and CYP450 enzymatic activity) [210]. Furthermore, Tanshinone IIA has beneficial effects on hepatic fibrosis via inactivation of hepatic stellate cells (HSCs) (at least via TGF-β1 and matrix protein decrease and apoptosis of HSCs increase), reducing AST, hyaluronic acid, MDA, and hydroxyproline, and decreasing the central markers of fibrosis, including collagen 1(α), tissue inhibitor of metalloproteinase (TIMP)-1, and α-smooth muscle actin (α-SMA) [210,211,212]. Additionally, the anti-HCC properties render the tashinone derivatives as potential factors, although their apoptosis-inducing effects may reduce the viability of hepatic cells, improving the HCC treatment.

**Figure 9:**
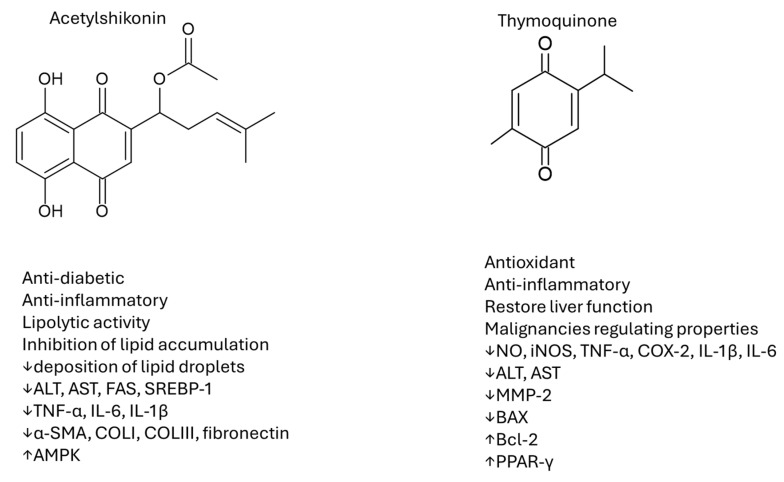
Effects of acetylshikonin and thymoquinone on NAFLD pathology.

Another quinone derivative is thymoquinone (TQ), the main bioactive component of black seed (*Nigella sativa*) oil, with antioxidant, anti-inflammatory and malignancy-regulating properties and efficacy in symptom alleviation of various inflammatory and metabolic disturbances [213]. It has also been shown to inhibit NO, iNOS, TNF-α and COX-2, IL-1β and IL-6 expression and production in LPS-stimulated hepatic cells and acute LPS/D-galactosamine (GalN)-induced hepatitis, affecting the transcription and activation of signaling factors such as activator protein (AP)-1, nuclear factor (NF)-κB and interleukin-1 receptor-associated kinase 1 (IRAK1) [214]. Apart from its general hepatoprotective effects, TQ has been shown to improve the progression of NAFLD in male Wistar rats given a high-fat high-cholesterol diet (HFCD) for 6 weeks (TQ was given 10 or 20 mg/kg for 6 weeks by oral gavage) [215]. The effects of TQ were dose-dependent, decreasing the ALT and AST levels up to 50% and 46%, respectively, with similar results for fasting blood glucose and insulin levels, leading to a normal homeostasis model assessment for insulin resistance (HOMA-IR) [index of insulin resistance: insulin (μU/mL) × glucose (nM)/22.5]. These results were followed by a significant decrease or increase accordingly, and in some cases, normalization of the levels of serum lipidemic indices (cholesterol, TG, HDL and non-HDL lipoproteins). This normalization of the diabetic and lipidemic markers, and the low oxidative content (MDA decreased by 58 and 73% in the two doses), may lead to substantial PPAR-γ liver gene expression. Furthermore, the anti-inflammatory activity of TQ was observed with a TNF-α decrease and an IL-10 increase, respectively. These cumulatively improved metabolic, oxidative and inflammatory characteristics may provoke the decreased apoptosis and fibrosis that was observed, since BAX was attenuated by 35% and 60%, and the anti-apoptotic Bcl-2 was increased significantly (1500%) in high doses of TQ, with matrix metalloproteinase-2 (MMP-2) and collagen fiber coloration decreased in the histological structure. TQ has also been shown to restore liver function after metabolic abnormalities induced by 6-propyl-2-thiouracil (PTU)-induced hypothyroidism, but at a much higher dose (400 mg/kg b.w. via intragastric intubation for six weeks) [216]. TQ could significantly increase the triidothyronine and tetraiodothyronine (T3 and T4) hormones levels, with a significant change in MDA, GSH, SOD and CAT serum levels (for CAT, a 12-fold increase in the liver levels was recorded), but not at GPx (that may be explained by the increase in the GSH levels), whilst an increase in the NO levels was also detected (NO production may be inflammatory, but in the case of low oxidative burden and inflammation, it is beneficial). The anti-inflammatory effect of TQ resulted in normalization of the lobular inflammation score, together with the NAFLD activity and the steatosis score, with no fatty degeneration or focal inflammatory reaction being observed by hematoxylin and eosin liver staining, and scarce CD68+ cells and alpha-smooth muscle actin reactions were observed. Cumulatively, these results accentuate the potential effects of TQ on the liver and metabolic and hormonal abnormalities, offering a spherical contribution to NAFLD treatment (Figure 9).

Emodin (EM, 6-methyl-1,3,8-trihydroxyanthraquinone) is a polyhydroxylated anthraquinone widely applied in Chinese medicine, with a wide range of pharmacological properties and applications, including antidiabetic, anticancer and cellular protective (hepatic and neuronal protection) [217]. However, disturbances of GSH and fatty acid metabolism in human liver cells, together with induction of apoptosis (implicating the mitochondria) in high doses, render its further analysis imperative [218,219]. In high-calorie laboratory chow, EM was tested at 40 mg/kg/d orally after induction for 12 weeks of the experimental NAFLD [219]. EM offered a substantial decrease in body weight, liver index, serum ALT, AST, TC, TG and hepatic TG, but no statistical significance in the change in the diabetes mellitus markers (fasting blood glucose, serum insulin and HOMA-IR). However, a remarkable decrease in liver mRNA levels of PPAR-γ was recorded, together with a pathological degree of liver steatosis. Despite the promising results, this study [220] had the drawback of using a high-caloric diet for the untreated rats and normal forage for the EM-treated group, perhaps accentuating the importance of the diet together with the administration of the compounds. In another study [221], EM (given at the same dose, 40 mg/kg/day orally) from the fifth week of a 15-week high-fat/high-fructose diet (HFD/HF) in Sprague Dawley rats significantly decreased the liver weight and index, considerably (with *p* ranging from 0.05 up to 0.01) the lipids (TG and TC) and the glucose insulin and HOMA-IR, but interestingly, caused a slight increase in body weight. These results were accompanied by improved cytological steatosis and ballooning, and absence of inflammatory cells, with normalization (similar to standard diet) of the plasma TNF-α and IL-6 levels. EM in primary hepatocytes from HFD/HF rats showed a decrease in the protein-bound GSH (ProSSG, oxidized GSH, similar to GSSG, with inflammation regulatory effects) and an increase in the total GSH (Tot GSH, improved ratio of protein-bound GSH/reduced GSH up to 80% compared to the non-treated group). Furthermore, emodin-treated HFD/HF primary hepatocytes could decrease the increased ratio of ProSSG/Tot GSH, provoked when the cells were cultured with hydrogen peroxide, and improve their viability, whilst co-culturing with N-acetylcysteine (NAC) offered remarkable improvement (significantly higher than NAC alone), resembling that of standard diet cells in ProSSG/Tot GSH and cell viability, and accentuating the role of EM in protecting from oxidative stress and its ability to offer synergistic results in the case of co-administration.

4-Acetylantroquinonol B (4-AAQB) is regarded as one of the major bioactive compounds in Androdia cinnamomea, structurally related to acetylated ubiquinone, with the ability to conduct oxidative and reductive reactions, and suppress the pro-inflammatory cytokine signaling cascade induced by inflammatory compounds such as lipopolysaccharides (LPS), which have been shown to result in liver inflammation and innate immune system activation [222,223]. 4-AAQB has been tested, pretreated in macrophage cell lines (RAW264.7 and J774A.1 cells), stimulated with LPS, and fed in a methionine/choline-deficient (MCD) diet (dose of 10 mg/kg intraperitoneally) to C57BL/6J mice [224]. 4-AAQB significantly attenuated MCD-induced NASH histological symptoms, steatosis and ballooning, immune cell filtration and serum ALT and AST (alanine and aspartate transaminase) levels (*p* was always lower than 0.05 and in many cases lower than 0.0001 compared to the non-treated group). Furthermore, significantly reduced LPS-induced NO production, with a decrease in the protein levels of inducible NO synthetase (iNOS), toll-like receptor-4 (TLR4), IKB-α, and signal transducer and activator of transcription 3 (STAT3), in liver tissues was recorded, signifying its effect on the amelioration of inflammatory responses. The anti-inflammatory and anti-apoptotic results include a decrease in the NLRP3 inflammasome, the apoptosis-associated speck-like protein and IL-1β and caspase-1. 4-AAQB also significantly decreased the protein level of pro-apoptotic BAX, the activation of c-Jun N-terminal kinase (JNK) and ER stress, with significant induction in the Nrf2 and SIRT1 levels in the liver, with a subsequent increase in HO-1 in the cell lines.

## 8. Alkaloids and Miscellaneous Derivatives

Berbamine (BBM) is a natural bis-benzylisoquinoline derived from Berberis-amurensis. Berbamine has been shown to aid the modulation of dysregulated pathways in several cancer types [225]. In vitro experiments have shown its role in reducing lipid accumulation and in ameliorating oxidative stress and proinflammatory responses [226]. Among these experiments, 0.5 mM of oleic acid was used to induce steatosis in HepG2 cells, which were treated afterwards with 1, 2, and 5 μM BBM [227]. BBM treatment resulted in decreased intracellular lipid concentration and improved hepatic cell morphology. BBM stimulated fatty acid β-oxidation through the activation of AMP-activated kinase (AMPK) and peroxisome proliferator-activated receptor (PPAR)-α and the restriction of SREBP-1c function [227]. Additionally, BBM treatment lowered oxidative stress and pro-inflammatory responses by significantly boosting antioxidant defenses in the liver and reducing NF-κB-regulated pro-inflammatory cytokines, such as TNF-α and IL-6. Furthermore, BBM treatment preserved mitochondrial function through the activation of the Nrf2/ARE pathway [227]. In this study, it was shown that berbamine treatment enhanced the nuclear accumulation of Nrf2, which notably upregulated the expression of HO-1, NQO-1, SOD2, and catalase. This increase in antioxidant enzyme levels effectively reduced the production of reactive oxygen species induced by OA-BSA and enhanced mitochondrial function. Thus, BBM could be considered a helpful agent in decreasing hepatic steatosis and managing oxidative stress, with its antioxidant and anti-inflammatory properties [227].

Demethyleneberberine (DMB) is a berberine (benzylisoquinoline alkaloid derivative) demethylated antioxidant derivative, with a wide array of activities ranging from antioxidant, anti-inflammatory, anticancer and decreasing ethanol-induced oxidative stress, intervening in iNOS, AMPK activation and improving mitochondrial dysfunction and steatosis in ALD [228]. In a similar prospect, DMB has been tested in HepG2 hepatocyte cultures and in male ICR mice [with methionine- and choline-deficient (MCD) high-fat diet for 4 weeks, DMB used intraperitoneally at 20 or 40 mg/kg once daily] [229]. DMB showed no cellular cytotoxicity, markedly decreased TG at the cellular level, with an increase in phosphorylated AMPK (dose- and time-dependently), ascertaining its AMPK-activating role. An in vivo significant reduction in liver TG, TC and serum ALT (up to 73 percent, reaching the levels of the control group) was followed by dose-dependent reversal of liver injury and lipid droplet (depicted with HE and Oil Red O staining) accumulation, with a parallel reduction in the liver/body index. Furthermore, a reduction in the oxidative stress was seen with normalization of the levels of GSH and MDA, and improvement in the relative mRNA levels of proteins (IL-1β, TNF-α, FAS, SREBP1c, PPAR-γ, PPAR-α, etc.) involved in inflammation, lipogenesis, steatosis, fibrosis and the promotion of NAFLD in the liver.

Bouchardatin is a naturally occurring β-indoloquinazoline alkaloid. It has been reported that bouchardatin exhibits a lipid-lowering function in adipocytes, as a novel inhibitor of lipogenesis [230]. Bouchardatin has also been shown to downregulate the expression of various transcription factors, such as CCAAT enhancer binding proteins (C/EBPβ, C/EBPδ, C/EBPα), peroxisome proliferator-activated receptors γ (PPARγ) and sterol-regulatory element binding protein-1c (SREBP-1c), which are regulators of lipogenesis [231,232]. These data showcase bouchardatin’s possible role in the management of steatosis in the context of NAFLD. In a study involving mice fed a high-fat diet to induce a NAFLD/NASH phenotype and HuH7 cell cultures, the effects of R17 (derivative of bouchardatin) regarding NAFLD were examined [233]. Administration of 20 mg/kg R17, i.p., every other day for 5 weeks reversed the induced increase in hepatic triglyceride content, reducing both triglyceride (TG) and diacylglycerol (DAG) levels. R17 also demonstrated anti-inflammatory effects by reducing macrophage infiltration in the liver and lowering plasma levels of inflammatory cytokines. Additionally, it alleviated liver injury, as evidenced by decreased plasma concentrations of alanine aminotransferase (ALT) and aspartate aminotransferase (AST), and inhibited liver fibrosis. In cultured cells, R17 reduced steatosis as well and mitigated oxidative stress by activating the liver kinase B1-AMP-activated protein kinase (AMPK) pathway through inhibition of ATP synthase activity [233]. Consequently, R17 and other substances derived from bouchardatin may have additive therapeutic potential in the management of NAFLD.

Another compound with intracellular reactive oxygen species downregulatory and paraoxonase-1 and Nrf2 activation-promoting effects is ankaflavin (AK), with an azaphilonoid structure. It has been shown to possess immunosuppressive properties, with a decrease in TNF-α, NF-κB and vascular cell adhesion molecule-1 (VCAM-1), together with triglycerides and cholesterol, preventing obesity and activating PPAR-γ [234,235]. AK could activate AMPK and suppress SREBP-1c (sterol regulatory element-binding protein-1c) and FAS expression in hepatocytes, decreasing the synthesis and uptake of fatty acids, cholesterol and triglycerides [236,237]. In the same direction, monascin, structurally similar to the AK compound and a secondary metabolite derived from Monascus-fermented products, showed the same activity with AK in oleic acid and a high-fat diet in FL83B hepatocytes and NAFLD in mice, with prevention of fatty acid accumulation in hepatocytes [236]. Both compounds promoted lipid metabolism (fatty acid beta-oxidation) with (PPAR)-α and AMPK activation, with significant attenuation in all the lipidemic markers, partially via farnesoid X receptor (FXR) and peroxisome proliferator-activated receptor gamma co-activator (PGC)-1α upregulation and carnitine palmitoyl transferase 1 (CPT-1), and an increase in acyl-CoA synthetase (ACS) and acyl-CoA oxidase (ACOX) levels. AK has also been shown to reduce the liver injury of ischemia-reperfusion (I/R) in a model of fatty liver mice, with I/R increasing the steatosis effects, whilst AK decreased hepatocyte apoptosis, inflammatory cytokines (TNF-α, IL-6 and IL-1β), serum aminotransferases and oxidative stress (lipid peroxidation inhibition), significantly decreasing the proliferation of Kupffer cells [238].

## 9. Limitations

This review has limitations that should be acknowledged. Firstly, the absence of a fully systematic and protocol-driven search strategy may have introduced selection bias and may limit the reproducibility of the findings. Although multiple databases were used, the narrative nature of the review inherently restricts the transparency of study selection. Additionally, the included studies are highly heterogeneous, ranging from in vitro experiments and animal models to a limited number of clinical trials (due to the rather low number of clinical findings), which constrains the ability to draw firm conclusions regarding clinical efficacy. Variability in study design, compound dosage, treatment duration, and experimental models (variation in the strain and type of animals and the applied experimental assay) further complicates direct comparisons across studies. Furthermore, while many natural compounds demonstrated promising antioxidant and anti-inflammatory effects, their comparison with established therapeutic approaches, such as insulin sensitizers, lipid-lowering agents, or vitamin E, remains limited or not applicable in the literature, making it difficult to position these compounds within current treatment regimens. Furthermore, critical aspects such as bioavailability, pharmacokinetics, and long-term safety are inconsistently addressed, thereby limiting their translational potential. Finally, the possibility of publication bias cannot be excluded, as studies reporting positive outcomes are more likely to be published. Collectively, these limitations highlight the need for cautious interpretation of the findings.

## 10. Future Perspectives

Future research should focus on strengthening the clinical translation of natural compounds in the management of NAFLD. Although preclinical studies have demonstrated promising antioxidant and anti-inflammatory effects in many cases, there is a clear need for well-designed, large-scale randomized controlled trials to confirm their efficacy and safety in human populations, as current evidence remains limited and sometimes inconsistent, in a rapidly expanding disease [239,240]. Standardization of dosage, formulation, and duration of treatment will be essential to improve comparability across studies and facilitate clinical application. Importantly, future studies should aim to directly compare natural compounds with emerging standard-of-care therapies, including recently approved or late-stage pharmacological agents such as thyroid hormone receptor-β agonists and GLP-1 receptor agonists, which have shown significant improvements in liver fat reduction and disease resolution in clinical trials [241]. Further investigations should also address the bioavailability and pharmacokinetics of these compounds, as many phytochemicals exhibit limited absorption and rapid metabolism, potentially restricting their therapeutic efficacy. Advances in drug delivery systems, including nanoformulations and targeted delivery strategies, may enhance their stability and biological activity [242,243,244,245]. In addition, fully elucidating the molecular mechanisms of action remains critical, particularly regarding pathways involved in lipid metabolism, insulin resistance, oxidative stress, and inflammation. Recent studies also highlight the importance of the gut–liver axis and microbiome modulation as promising therapeutic targets in NAFLD [246].

Finally, future research should also explore the integration of natural compounds into precision medicine approaches, enabling the identification of patient subgroups most likely to benefit from such interventions. Given the rapidly evolving therapeutic landscape and the development of novel pharmacological agents currently under clinical investigation, natural compounds may ultimately serve as complementary or adjunctive therapies rather than standalone treatments, contributing to a more holistic and multi-targeted strategy for NAFLD management in an area that is currently lacking targeted therapies [247,248,249,250].

## 11. Conclusions

This review analyzed supplementary compounds derived from natural products that treat clinical conditions associated with NAFLD and reduce oxidative stress and inflammatory activity during disease progression, as they are the major factors of the pathogenesis. Most of these compounds were able to reduce or prevent the progression of NAFLD by improving metabolic and hepatic factors, such as liver enzyme levels, lipid biosynthesis, and insulin resistance (IR). They have also been found to modulate inflammatory and lipotropic activity, such as AMPK/SIRT1 activation, and downregulation of PPARγ, NF-κB, and MAPK, suppressing lipogenesis and contributing significantly to the reduction of inflammation and oxidative stress. Furthermore, multi-target compounds are an important array of compounds, as they can serve as effective therapies, alone or in combination with other compounds, for the progression of NAFLD by blocking multiple disease pathways through different mechanisms, offering improved clinical and histopathological outcomes. In conclusion, the reported drugs and compounds showed beneficial activity on NAFLD; however, many limitations still arise, and more studies are needed to determine the efficacy and toxicity of these products, especially at the clinical level. These results indicate that the referred compounds are useful for potential treatment or disease amelioration and may also be able to be used as lead compounds for the design of new molecules with improved characteristics.

## Data Availability

No new data were created or analyzed in this study. Data sharing is not applicable to this article.

## Abbreviations

ABCA1: ATP-binding cassette transporter A1; ACC: acetyl-CoA carboxylase; ACC1: acetyl-CoA carboxylase 1; ACOX1: acyl-CoA oxidase 1; Acta-2: Actin Alpha 2; AGEs: advanced glycation end-products; AKT (PKB): protein kinase B; ALD: alcohol-related liver disease; Alox5: arachidonate 5-lipoxygenase; ALT: alanine aminotransferase; AMPK: AMP-activated protein kinase; AP: activator protein; AST: aspartate aminotransferase; ATF4: activating transcription factor 4; ATGL: adipose triglyceride lipase; ATP: adenosine triphosphate; BMI: body mass index; cAMP: cyclic adenosine monophosphate; CAT: catalase; CHOP: C/EBP homologous protein; CHO: cholesterol; CK7: cy-tokeratin 7; COL1A1: collagen type I alpha 1 chain; COX-2: cyclooxygenase 2; CPT1A: carnitine palmitoyltransferase 1A; CPT1B: carnitine palmitoyltransferase 1B; CYP: cytochrome P450; DEN: diethylnitrosamine; DGAT1: diacyl-glycerol acyltransferase 1; DGAT2: diacylglycerol acyltransferase 2; DNA: deoxyribonucleic acid; DNL: de novo lipogenesis; eIF2α: eukaryotic initiation factor 2 alpha; eNOS: endothelial nitric oxide synthase; ER: endoplasmic reticulum; ERK1/2: extracellular signal-regulated kinases 1/2; FAS: fatty acid synthase; FASN: fatty acid synthase; FFAs: free fatty acids; FGF: fibroblast growth factor; FXR: farnesoid X receptor; G6Pase: glucose-6-phosphatase; GPx: glutathione peroxidase; GRP78: glucose-regulated protein 78; GSH: glutathione; GSK3β: glycogen synthase kinase 3 beta; GSSG: oxidized glutathione; GSTA1: glutathione S-transferase A1; GTT: glucose tolerance test; H2O2: hydrogen peroxide; HAT: histone acetyltransferase; HCC: hepatocellular carcinoma; HDL: high-density lipoprotein; HF: high-fructose; HFHC: high-fat, high-cholesterol; HFD: high-fat diet; HIF-1: hypoxia-inducible factor 1; HMGB1: high mobility group box 1 protein; HMGCR: 3-hydroxy-3-methylglutaryl-coenzyme A reductase; HO-1: heme oxygenase 1; HOMA-IR: homeostatic model assessment of insulin resistance; HSCs: hepatic stellate cells; HSL: hormone-sensitive lipase; hs-CRP: high-sensitivity C-reactive protein; IKK: inhibitor of kappa B kinase; IL-1β: interleukin 1 beta; IL-4: interleukin 4; IL-10: interleukin 10; IL-18: interleukin 18; IR: insulin resistance; I/R: ischemia–reperfusion; IRAK1: interleukin-1 receptor-associated kinase 1; IRE1: inositol-requiring enzyme 1; IRS-1: insulin receptor substrate 1; ITT: insulin tolerance test; JNK: c-Jun N-terminal kinase; LDL: low-density lipoprotein; LOX: lipoxygenase; LPL: lipo-protein lipase; LPS: lipopolysaccharide; LXRs: liver X receptors; MAFLD: metabolic dysfunction-associated fatty liver disease; MAPK: mitogen-activated protein kinase; MASLD: metabolic dysfunction-associated steatotic liver disease; MCD: methionine- and choline-deficient diet; MCP-1: monocyte chemoattractant protein 1; MDA: malondialdehyde; MUC1: mucin 1; MUC2: mucin 2; NAFLD: non-alcoholic fatty liver disease; NADPH: nicotinamide adenine dinu-cleotide phosphate; NF-κB: nuclear factor kappa B; NLRP3: NOD-like receptor family pyrin domain containing 3; NLRs: nucleotide-binding oligomerization domain-like receptors; NO: nitric oxide; NOX: NADPH oxidases; NOX1: NADPH oxidase 1; NQO1: NAD(P)H quinone dehydrogenase 1; NRF2: nuclear factor erythroid 2-related factor 2; PAP: phosphatidate phosphatase; PEPCK: phosphoenolpyruvate carboxykinase; PERK: protein kinase RNA-like ER kinase; PGC-1α: peroxisome proliferator-activated receptor gamma coactivator 1 alpha; PGC-1β: peroxisome pro-liferator-activated receptor gamma coactivator 1 beta; PDI: protein disulfide isomerase; PI3K: phosphatidylinositol 3-kinase; PPARα: peroxisome proliferator-activated receptor alpha; PPARγ: peroxisome proliferator-activated re-ceptor gamma; PRRs: pattern recognition receptors; PTP1B: protein tyrosine phosphatase 1B; PTU: propylthiouracil; RAGE: receptor for advanced glycation end-products; REG3γ: regenerating islet-derived protein 3 gamma; RELMβ: resistin-like molecule beta; ROS: reactive oxygen species; SCD1: stearoyl-CoA desaturase 1; SFRP1: secreted friz-zled-related protein 1; SKQ1: decyltriphenylphosphonium; SOD: superoxide dismutase; SQSTM1: sequestosome 1; SREBP: sterol regulatory element-binding protein; SREBF1: sterol regulatory element-binding transcription factor 1; STAT5: signal transducer and activator of transcription 5; STAM: Stelic animal model; T2DM: type 2 diabetes mellitus; TAA: thioacetamide; TAC: total antioxidant capacity; TBARS: thiobarbituric acid-reactive substances; TFF3: trefoil factor 3; TG: triglycerides; TGF-β1: transforming growth factor beta 1; TIMP-1: tissue inhibitor of metalloproteinases 1; TLRs: toll-like receptors; TNF-α: tumor necrosis factor alpha; UCP2: uncoupling protein 2; UPR: unfolded protein response; XBP-1: X-box binding protein 1; 4-HNE: 4-hydroxynonenal; α-SMA: Alpha Smooth Muscle Actin.
